# Wheat and Rice beyond Phenolic Acids: Genetics, Identification Database, Antioxidant Properties, and Potential Health Effects

**DOI:** 10.3390/plants11233283

**Published:** 2022-11-29

**Authors:** Fereidoon Shahidi, Renan Danielski, Samantha Ottani Rhein, Lee A. Meisel, Jocelyn Fuentes, Hernan Speisky, Andrés R. Schwember, Adriano Costa de Camargo

**Affiliations:** 1 Department of Biochemistry, Memorial University of Newfoundland, St. John’s, NL A1C 5S7, Canada; 2 Nutrition and Food Technology Institute, University of Chile, Santiago 7830490, Chile; 3 Departament of Plant Sciences, Facultad de Agronomía e Ingeniería Forestal, Pontificia Universidad Católica de Chile, Santiago 7820436, Chile

**Keywords:** cereals, by-products, phenolic antioxidants, flavonoids, anthocyanins, proanthocyanidins

## Abstract

Wheat and rice play a vital role in human nutrition and food security. A better understanding of the potential health benefits associated with consuming these cereals, combined with studies by plant scientists and food chemists to view the entire food value chain from the field, pre and post-harvest processing, and subsequent “fork” consumption, may provide the necessary tools to optimize wheat and rice production towards the goal of better human health improvement and food security, providing tools to better adapt to the challenges associated with climate change. Since the available literature usually focuses on only one food chain segment, this narrative review was designed to address the identities and concentration of phenolics of these cereal crops from a farm-to-fork perspective. Wheat and rice genetics, phenolic databases, antioxidant properties, and potential health effects are summarized. These cereals contain much more than phenolic acids, having significant concentrations of flavonoids (including anthocyanins) and proanthocyanidins in a cultivar-dependent manner. Their potential health benefits in vitro have been extensively studied. According to a number of in vivo studies, consumption of whole wheat, wheat bran, whole rice, and rice bran may be strategies to improve health. Likewise, anthocyanin-rich cultivars have shown to be very promising as functional foods

## 1. Introduction

Wheat was first domesticated ~10,000 years ago in the boomerang-shaped Middle Eastern region of the Fertile Crescent, a region rich in anthropological evidence of early human civilizations [1]. The earliest cultivated types of wheat were hulled species containing tough glumes, which are removed by threshing. These early cultivated wheat species were the diploid Einkorn (*Triticum monococcum* L. subsp. *monococcum* (AA) and the tetraploid Emmer (*T. turgidum* spp. *dicoccum* (AABB) [2]. Although both species spread to Europe about 9000 years ago, they are now rarely cultivated and are now only found growing in mountainous areas of central and eastern Europe, Turkey, Caucasus, and Morocco [3].

Free-threshing, glume-free forms of wheat also evolved during this period, including tetraploid and hexaploid common (bread) wheat. Common wheat (*T. aestivum*, AABBDD) was formed through the accidental hybridization of Emmer with the wild wheat species *T. tauschii* [4]. Of various minor species of wheat, the most important is spelt (*T. aestivum* var. *spelta* or *T. spelta* [AABBDD]), a hulled species closely related to common wheat, widely cultivated in central Europe about 4000 years ago [5]. Wild emmer (*T. dicoccoides*, AABB) likely arose first from a relative of wild Einkorn (*T. urartu*, AA) and presumably wild spelt (*Aegilops speltoides*, BB or SS). Spelt and common wheat finally arose through hybridization with another wild wheat, *T. tauschii* (DD) [2,4].

The world production of wheat is currently around 760.9 million tons, associated with 219.0 million hectares grown globally, with average yields of 3.5 t/ha in 2020, and is presently one of the major crops cultivated in the world, in which China, India, Russia, United States, and France are the main producers [6]. According to the June 2022 FAO Food Outlook report [7], the global wheat market has faced much uncertainty during the 2022/2023 season due to the ongoing war in Ukraine, trade policy changes in several countries, and high international prices. Drought conditions in the northern hemisphere have negatively affected wheat production and yields. There is a significant forecast for a decline in world wheat production in 2022/2023.

The wild ancestor of rice, *Oryza rufipogon*, grows within Southern Asia, and collections of early domesticated rice in this region may originate from harvesting wild seeds [8]. The earliest dated domesticated rice *(O. sativa japonica*) was grown as early as 8000 BP within the Yangtze River valley of southern China. However, according to genetic and archeological evidence, nonshattering (a key domestication trait) [9] was not selected for another thousand years [10]. Genetic evidence suggests that the two main rice cultivars, *japonica* and *indica*, may have been independently domesticated [11,12]. In this context, rice was also grown in India at about 5000 BP; although, the domesticated *indica* subspecies presently seem to be a result of the incorporation of favorable alleles from *japonica* [10] or wild populations [13]. *Indica* was domesticated in the southern area of the Himalayan mountain range, probably in eastern India, Thailand, and Myanmar [11]. Rice reached other parts of India approximately 4500 years ago and Japan about 2400 years ago [6]. Globally, it is currently cultivated on around 164.2 million ha with an annual production of 756.7 million tons, and the world yield average was about 4.6 t/ha in 2020 [6]. The top rice-producing countries in the world are China, India, Indonesia, Bangladesh, and Vietnam [6].

Wheat and rice contain phytochemicals that protect them against abiotic and biotic stresses but also may provide health benefits by reducing the risk of chronic diseases, which is supported by studies in vitro and in vivo. The available literature usually focuses on only one food chain segment. Therefore, there is a literature gap for inter/multidisciplinary groups that involve plant scientists, agronomists, food scientists, and other players involved in the wheat and rice production chain, including the industry. Therefore, to fill this gap, this article aims to present the first narrative review focusing on the potential health benefits of polyphenols from wheat and rice from a perspective that involves plant genetics, phenolic identification database, antioxidant properties, and health beyond ferulic and other phenolic acids in both wheat and rice. Therefore, by summarizing the information on two of the most important cereals for human consumption, this novel compilation will be useful for plant scientists, agronomists, food scientists, and many other players involved in the wheat and rice production chain from the farm to the fork and beyond. Finally, this work may influence future advances in the field by setting some future perspectives.

## 2. Wheat and Rice as Sources of Phenolic Antioxidants

Eating whole-grain cereals has been associated with a decreased risk of developing chronic ailments such as diabetes [14], obesity [15], cancer, and cardiovascular diseases (CVDs) [16]. These benefits were associated with the fiber content in whole grains for a long time. However, recent reports suggest that, in reality, these health benefits result from a combination of dietary fiber and other bioactive components, mainly phenolic compounds. Phenolic acids, monomeric flavonoids, and tannins are the dominant bioactive phenolics in cereal grains [17].

Phenolic acids are biosynthesized from the amino acid phenylalanine using the phenylpropanoid pathway or, to a lesser extent, from tyrosine. They can be grouped into hydroxycinnamic (e.g., *p*-coumaric, caffeic, ferulic, and sinapic) and hydroxybenzoic (e.g., *p*-hydroxybenzoic, vanillic, syringic, and gallic) acids, based on their C1-C6 and C3-C6 skeletons, respectively. In cereal grains, phenolic acids are encountered as free, soluble conjugates (esterified and etherified forms) and insoluble-bound forms [18,19].

Flavonoids are among the largest phenolic group of compounds in nature. Usually, they consist of two aromatic rings linked to a heterocyclic ring through a carbon bridge. One or more hydroxyl groups are connected to the aromatic ring. Like phenolic acids, the number and the position of hydroxyl groups play an important role in the antioxidant activity of a particular flavonoid. Some grain species contain anthocyanins, a group of water-soluble flavonoid pigments that, depending on the pH, may exhibit a red, purple, blue, or black color [18,20,21]. Generally, anthocyanins are purple in neutral pH, assuming red under acidic conditions, and shift to blue in increasingly basic pH [22]. Anthocyanins, red-colored pigments predominantly flavylium cations, can play an important role in the sensory properties of functional foods [23].

Tannins have been defined as water-soluble polyphenols bearing a molecular weight between 500 and 3000 D. These natural compounds are also known for presenting a significant number of hydroxyl or other functional groups (1 to 2 per 100 D). Therefore, tannins are mainly present in food as large polymerized molecules, which can be formed either by the plants themselves or by food processing. These compounds can bond with sugars and dietary proteins to create glycosides and polyphenolic proteins, respectively [18]. In summary, tannins are divided into hydrolyzable tannins (esters of gallic or ellagic acid, gallotannins, and ellagitannins) [24,25] and condensed tannins (proanthocyanidins).

Hydrolyzable tannins are made of a polyhydric alcohol central core, such as glucose, and hydroxyl groups. The hydroxyl groups are esterified by gallic acid (gallotannins) or hexahydroxydiphenic acid (ellagitannins). Glucose and gallic acid are derived from the hydrolysis of gallotannins with acids, bases, or specific enzymes. Whereas ellagic acid is formed by the lactonization of the hexahydroxydiphenic acid of ellagi-tannins.

Proanthocyanidins are dimers, oligomers, or polymers of flavan-3-ol and flavan-3,4-diols or a mixture of them bound through carbon-carbon and ether linkages. Oligomeric proanthocyanidins with two to ten degrees of polymerization, while polymers have greater than 10 degrees of polymerization [20]. Within the proanthocyanidins, procyanidins with a (epi)catechin as their basic units, are the most important in plant foods [18,26]; while prodelphinidins and propelargonidin (which have (epi)gallocatechin and (epi)afzelechins as their respective basic units) may also be found [26,27]. To specifically indicate where each of these phenolic classes is located in the cereal grain, it is necessary to understand its anatomy [28].

Whole grains consist of hull, bran, endosperm, and germ. The hull is the inedible outer layer, and its function is to protect the inner part of the grain from environmental factors (e.g., sunlight, pests, water, and diseases). One can find the bran below the hull, which is a multi-layered outer skin often ground into bran flour [17]. The bran is rich in fiber, iron, zinc, copper, magnesium, B vitamins, and phenolic compounds [17,29,30]. The endosperm makes up the most substantial portion of the grain, and its function is to protect and provide nutrition to the germ. This edible part, generally commercialized as pearled or polished grain, concentrates carbohydrates, proteins, and small amounts of vitamins and minerals. Finally, the germ (also called the embryo) is the part that sprouts into a new plant. The germ also contains vitamins E (mostly alfa-tocopherol) [31], B1, B2, and B6 [32], and phenolics [17].

During the milling of rice, the endosperm (edible portion) is separated from the other fractions, which account for approximately 30% of the grain’s weight, namely, husk (20%), bran (8%), and germ (2%). However, the exclusion of such by-products is undesirable from a nutritional point of view. The husk, the bran, and the germ are important sources of phenolic bioactives and other important minor compounds such as phytosterols, tocopherols, tocotrienols, phytic acid, and γ-oryzanol [33]. The same can be said for wheat. Consuming 2–3 servings of whole grains (around 48 g) per day may decrease the relative risk of developing certain forms of cancer, type 2 diabetes, and cardiovascular ailments [34,35,36,37,38]. This decrease may be related to the diversity of bioactive compounds in the bran and germ, which are generally discarded or destined for animal feed after grain refining. For instance, wheat bran, which corresponds to 13–17% of the grain’s composition, is rich in insoluble-bound phenolic acids, such as ferulic, *p*-coumaric, and sinapic acids [37]. The concentration of these components is influenced by growing conditions [17,39], but some trends are easily noted. Red and black rice, for example, are reported to have a higher phenolic antioxidant content than white rice [40].

The bran renders the highest contribution to the phenolic content of rice [17], which, as mentioned earlier, is present in both soluble (free, esterified, and etherified) and insoluble-bound forms [41,42]. This classification is essential as the phenolic form defines their bioefficiency, a combination of bioaccessibility and bioavailability, as discussed in Section 5 of this article. An updated phenolic database is important for a better understanding of their bioefficiency. It may help explain the relationship between the regular consumption of refined and whole grains and the potential use of their by-products to develop nutraceuticals to reduce the risk of chronic diseases. Phenolic databases are also crucial for decision makers to suggest ideal intake levels so a particular compound or a feedstock containing sufficient levels of a well-characterized compound can provide health benefits under physiological conditions. The following section will revise the phenolic composition of selected rice and wheat cultivars and by-products thereof and discuss their potential as antioxidants and their bioefficiency.

## 3. Genetics

Comparative analysis of phenolic acid content in different wheat genetic backgrounds [43,44,45,46,47,48,49] suggests that individual and total phenolic acid levels are complex traits affected by genotypic, environmental, and “genotype × environment” interactions [50,51]. One study on tetraploid wheat reported a higher proportion of genotypic variability to total variability for both total and individual phenolic acid content, suggesting that breeding programs may enhance these traits [52]. Recently, one genome-wide association study (GWAS) using tetraploid wheat identified 22 quantitative trait loci (QTL) associated with the synthesis of various phenolic acid compounds [51]. In addition, these authors detected two QTL for *p*-coumaric acid that coincided with the *p*-coumarate 3-hydroxylase (C3H) and the phenylalanine ammonia-lyase (PAL2) genes. These gene-based single nucleotide polymorphisms (SNPs) markers may help unravel the mechanisms by which phenolic acids accumulate in wheat grains, potentially leading to the bioactivity enrichment of wheat end-products.

In this same context, Sharma et al. [48] identified 44 different phenolic biosynthetic genes that were differentially expressed during grain filling using microarray analyses (17 confirmed with qRT-PCR). These genes had a low expression level in a good quality chapatti (unleaved flat bread) cultivar. In contrast, 12 genes were differentially expressed when comparing a good chapatti quality cultivar with a poorer one. These differentially expressed genes may be suitable candidate genes for common (bread) wheat molecular marker-based breeding programs. Ferulic acid is the most abundant phenolic compound at all wheat developmental phases, constituting 82–92% of the total phenolic content (TPC) [53,54]. In this sense, the expression of nine phenolic biosynthetic genes (TaC3H1, TaC3H2, TaC4H, TaCOMT1, TaCOMT2, TaPAL1, TaPAL2, Ta4CL1, and TACL2) were studied during common wheat grain filling through qRT-PCR analyses. Peak ferulic acid levels were detected 14 days post-anthesis, and the genes associated with its biosynthesis showed three different expression patterns. Higher phenolic acid levels and elevated expression of related genes were detected at the beginning of grain filling in white wheat. In contrast, the highest levels were seen at a later stage of development in purple wheat [55], suggesting a strong correlation between phenolic acid accumulation and the expression of phenolic acid biosynthetic genes. In this context, wheat grains’ pigmentation (anthocyanins and flavonoids) is a breeding trait that may be associated with increased bioactive compounds in seeds and subsequent food products [49].

Consuming mycotoxin-contaminated feedstocks may induce detrimental health effects in humans and animals. Therefore, minimizing human exposure to contaminated wheat is critical [56,57]. Accordingly, another well-investigated property of ferulic acid is its potent inhibition of mycotoxin synthesis of many Fusarium strains, particularly, type B trichothecene [58]. qRT-PCR analyses demonstrated a decline in the transcript levels of Tri genes in response to ferulic acid, suggesting a transcriptional inhibition of toxin synthesis by ferulic acid. In addition, 8-5′-benzofuran dehydrodiferulic acid (an abundant dimeric form of ferulic acid) inhibited trichothecene biosynthesis similarly to the monomer form [59]. Another study [60] concluded that the resistance in Fhb1 (a Fusarium head blight resistance QTL on 3BS) was principally related to increased cell wall thickening due to the accumulation of hydroxycinnamic acid amides, flavonoids, and phenolic glucosides [60].

The transgenic expression of barley, wheat, and rice HGGT (homogentisic acid geranylgeranyl transferase) in *Arabidopsis thaliana* leaves and maize seeds increased the total vitamin E antioxidants, tocopherols, and tocotrienols, relative to non-transformed plants [61]. In addition, 4-hydroxyphenylpyruvate dioxygenase-overexpressing transgenic common wheat plants exhibited a 2.4-fold increase in tocochromanol levels, which is potentially valuable because of its antioxidative and nutritional properties [62]. For the interest of plant breeders, significant variations in vitamin E have been reported in several agronomic crops of economic relevance [63], including rice [64], canola [65], and in maize GWAS analyses [66,67,68]. These studies show how GWAS detects QTL and candidate genes to identify components for new metabolic engineering strategies for biofortification, which are valuable to breed vitamin-rich food crops [69].

Gramineous cell walls are composed of the important matrix polysaccharides, type II walls containing β-glucans and arabinoxylans, and arabinoxylans with ferulate residues that are ester-bound to the hemicellulosic fraction, glucuronoarabinoxylan (GAX) [70,71]. Ferulic acid is subjected to a coupling reaction that forms diferulic acid (DFA) that cross-link arabinoxylan molecules [72]. Consequently, the synthesis of arabinoxylan-DFA cross-links leads to a firm complex inside the architecture of the cell wall [73,74], with ferulic acid having a structural function within the cell wall. Grasses have reduced feruloylation of GAX in their cell walls, making them easy to transform into biofuel, which is vital for biomass processing [75]. In this previous study, the BAHD acyl-coA transferase superfamily genes and the rice *p*-coumarate monolignol transferase (*OsPMT*) gene encoded proteins mainly associated with bound (*p*CA), and other BAHD candidates were controlling for bounding ferulic acid. Another study reported that the *OsAt10* gene, a BAHD acyltransferase, is involved in GAX modification, and overexpression of *OsAT10-D1* was associated with 20–40% in rice saccharification yield; *OsAt10* being a valuable target for enhancing grass cell wall quality for animal feed and fuel [76]. Other genes studied have been cell wall-bound peroxidase (CW-PRX) and phenylalanine ammonia-lyase (PAL), whose increases in transcript expression and activities were cooperatively implicated in the arrangement of the ferulate network of the cell walls of the rice shoots. These genes can be partially related to their enzymatic actions, and their increased expression and activity levels in such a network are likely responsible for the cell wall maturation, resulting in the ending of the elongation growth of coleoptiles [77]. Another research work reported that two contrasting drought-tolerance rice cultivars were subjected to transcriptomic and metabolomic studies using drought and well-watered conditions, and 4-hydroxycinnamic acid (*p*-coumaric acid) and ferulic acid were essential metabolites for rice drought-tolerance [78]. The authors concluded that well-maintained photosynthesis under drought enhances rice drought-tolerance, and specific metabolites are vital to protecting photosynthesis and dehydration through antioxidant mechanisms or osmotic adjustment. Another study showed transcriptomic responses to rice roots exposed to three rhizotoxic environmental stresses (mercury, chromium, and ferulic acid) associated with early-transient, early-constant, and delayed gene inductions [79]. Network component analyses involved intricate connections among common genes, standing out as the most highly connected signaling hubs encompassing leucine-rich repeat receptor-like kinase (LRR-RLK), non-expressor pathogenesis-related 1 (NPR1), mitogen-activated protein kinase 5 (MAPK5), and protein phosphatase 2C 68 (PP2C68). These authors concluded that properly adjusted transcriptional regulation in response to environmental stress in rice depends upon signal intensity and duration and genomic architecture.

In rice, two types of regulatory genes work as transcriptional stimulators: (a) the *C1/Pl* gene family is responsible for the synthesis of proteins with homology to the DNA-binding domain of MYB-like proteins, and (b) the *R/B* gene family produces proteins with homology to the basic helix–loop–helix (bHLH) region of MYC-like proteins [80]. According to these authors, two families synthesize related proteins with distinct expression models, highlighting that the crosstalk between regulatory enzymes and proteins involved in the polyphenol biosynthetic route triggers the coloring of different rice organs. The combination of the *R* and *C1* genes controls the kernel color, whereas the combination of the *Pl* and *B* genes regulates the pigmentation of mature rice tissues, such as the leaves and the husk. Genetic studies have identified two loci, *Pp (Prp-a)* and *Pb (Prp-b)*, which are positioned on chromosomes 1 and 4, respectively, which regulate the anthocyanin coloring of the black rice seed coat. However, additional characterization and functional confirmation must be carried out to confirm these roles.

*Rd* (red pericarp and seed coat) and *Rc* (brown pericarp and seed coat) jointly generate red pericarp pigmentation. *Rc* alone results in brown pigmentation of seeds, whereas Rd by itself associates with no specific phenotype [81]. The *Rc* alleles that have been cloned to date are as follows: (a) *Rc-s*, which relates to light red color; (b) *Rc*, which generates brown stains on a reddish-brown background; and *rc*, a null allele [81,82]. These last authors cloned *Rd*, which produces dihydroflavonol 4-reductase. Using one microarray, Kim et al. [83] identified 82 transcription factors that may be related to anthocyanin synthesis in black rice and discovered 12 putative genes by comparing two black cultivars with one white cultivar. In addition, these authors also identified 15 genes that may be implicated in the anthocyanin metabolic pathway of the polyphenols biosynthesis. However, the specific functions of these genes and transcription factors must be deciphered in future studies. More recently, a GWAS study associated whole grain color (red and white) with antioxidant capacity and phenolic profiles (free and bound) [84]. The *Rc* gene was associated with all free phenolics and antioxidant activity. Three loci for five phenolic-related characters or more were also reported; two loci for two free phenolic-related traits or more were detected, as well as one locus for ferulic acid. These loci may provide insight into potential candidate genes for cloning, which is valuable for understanding the functional genetics regulating the phenolic acid biosynthesis in rice.

Some evidence suggests that rice cell wall strengthening for disease resistance and allelopathy may correlate with phenylamides. Phytopathogen attacks and abiotic stimuli involving UV radiation promote the synthesis of phenolic phytoalexins [85]. These authors showed evidence that the arylmonoamine, shikimate, and phenylpropanoid pathways are fine-tuned for phenolic phytoalexin synthesis involving genes regulated by abiotic and biotic stresses in rice. For example, broad-spectrum resistance to blast fungus (*Magnaporthe oryzae*) is modulated by one rice gene, *OsBBI1*, which codes for a RING finger protein with E3 ligase and alters cell wall defense mechanisms [86]. These authors functionally characterized *OsBBI1*, generating knowledge of its E3 ligase-mediated innate immunity as a potential strategy for creating broad-spectrum resistance against this devastating rice disease.

Regarding bacterial diseases, Fan et al. [87] identified different phenolic substances that suppressed the virulence of a very destructive bacterial infection in rice, *Xanthomonas oryzae*, using the type III secretion system (T3SSs). In this study, ten (including *o*-coumaric acid) out of 56 phenolic compounds considerably hindered the promoter activity of a harpin gene, *hpa1*, significantly reducing the hypersensitive response (HR) caused by this bacteria. In parallel, qRT-PCR studies of the genes on the *hrp* (hypersensitive response and pathogenicity) cluster and the regulatory genes *hrpG* and *hrpX*, decreased their expression as a result of the application of four inhibitors, indicating that the bacteria T3SS expression was suppressed; however, other virulence factors were not affected, suggesting a probable T3SS-specific inhibition. Another gene thoroughly studied in rice is *OsWRKY89*, a member of the large WRKY transcription factor family of proteins [88]. In this study, the authors functionally characterized *OsWRKY89* and performed transcriptional, RNAi, RT-PCR, and HPLC experiments by overexpressing the *OsWRKY89* gene, which improved resistance to white-backed planthopper and the rice blast fungus, and it increased the UV-B irradiation tolerance. Park et al. [89] identified regulatory networks involving transcription factors, calcium signaling compounds, G-proteins, signal-perceiving receptor kinases, and the signal-transducing mitogen-activated protein kinases, which regulate phytoalexin and flavonoid biosynthesis in UV-C stressed rice leaves. In this context, Mohanty et al. [90] reported crosstalk between distinct plant hormones and the participation of essential transcription factors such as ERF (jasmonic acid-inducible), MYB, ZnF, bHLH, WRKY, bZIP, in the control of higher phenolic compound and carotenoid accumulation in response to blue light. This study revealed that the light-specific regulatory mechanisms implicated in synthesizing these compounds with critical interconnections between these transcription factors are essential in rice leaves.

Allelopathy is a promoting and repressive reciprocal biochemical interconnection between plants and microorganisms. A great assortment of chemicals, including phenolic acids, detected in rice root exudates and decomposing rice leftovers, are putative allelochemicals that can interrelate with the adjacent environment, which affect weeds, soil insects and diseases, microbial diversity around rice plants, including soil characteristics [91]. Considering that these allelopathic interactions may be beneficial, these authors concluded that the development of crops with improved allelopathic features by genetic modification must be carried out with extreme care, considering the ecological risk evaluation (i.e., crop yields, the ratio of cost and benefit, non-hazardous and safe for the ecosystem and humans, among others). Zhang et al. [92,93] triggered rice allelopathy with Barnyard grass root exudates, up-regulating the phenylalanine ammonia-lyase (*PAL*) gene in allelopathic rice (PI312777) leaves. Transcriptomic analyses confirmed that rice allelopathy is a chemical initiation process and a viable and practical tool to improve this crop.

## 4. Phenolics of Rice, Wheat, and Their Processing By-Products

Quantification of TPC is a term that has been used for a long time [94]. Traditionally, this quantification was determined using the traditional Folin–Ciocalteu reagent. However, non-phenolic compounds may react reductively with this reagent [95]. An example of this was seen in the analysis of milk (devoid of phenolic extracts) with this reagent, which resulted in a non-zero phenolic content. This variability may have resulted from milk proteins (e.g., tyrosine residues) and sugar components (oligosaccharide and glucose) interfering with the reagent. Another limitation of using this reagent to quantify TPC is the variable reactivity of different compounds to the reagent. Therefore, a single numerical expression of the results of these analyses is arbitrary [95], and the results should be expressed as a comparative trend of several samples rather than one. These analyses are standard in screening wheat and rice samples in breeding programs to identify elite genotypes with the highest TPC. In this sense, TPC provides an index or trend rather than a precise quantification.

**Figure 1:**
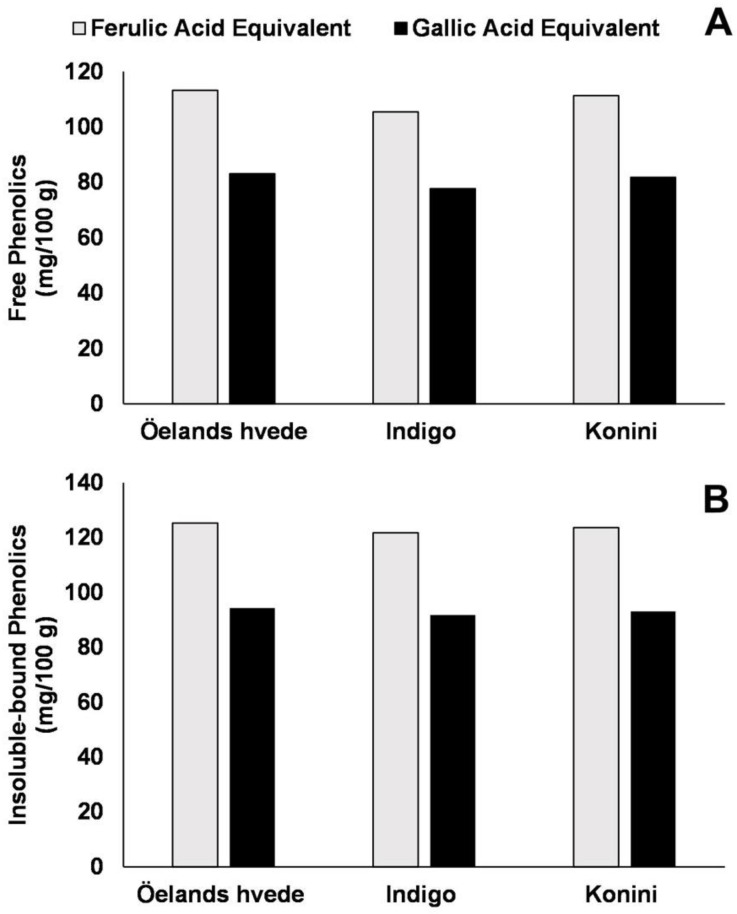
Total phenolic content of free (**A**) and insoluble-bound (**B**) fractions of wheat flour from common and purple wheat grains. Adapted from Yu and Beta [96].

As mentioned earlier, due to varying redox potentials, there is a variation in the reactivity of specific phenolic molecules to the Folin–Ciocalteu reagent. The TPC is highly influenced by the standard used to calculate the results. According to data published by Yu and Beta [96], the content of free and insoluble-bound phenolics from wheat flour was up to 36 and 33%, respectively, slightly higher when the results were calculated in terms of ferulic acid equivalents instead of gallic acid equivalents (Figure 1). Therefore, the term quantification should be read carefully and probably be replaced by the term “estimation,” as done by some authors [97]. The word “estimation” gives an accurate idea of the uncertainties associated with this type of method since several other factors influence the quantification, such as the solvent system used to recover soluble phenolics [98], the technique used to release insoluble-bound compounds (chemical or enzymatic hydrolysis), the effects of solvent to solid-ratio, particle size, and time of extraction. In summary, the term estimation would also imply that in-depth analysis (e.g., hyphenated techniques such as liquid chromatography coupled to tandem mass spectrometry—tandem mass spectrometry) must be carried out.

Information on the presence of phenolic compounds in cereals and their processing by-products is not new. Studies from the seventies have already addressed the identification of phenolic acids (e.g., ferulic, caffeic, vanillic, *p*-coumaric, sinapic, and salicylic acid) in wheat and rice [99,100,101]. Since then, analytical techniques have significantly improved, allowing the conduction of more sophisticated analyses, which has made it possible to advance the knowledge and gain a detailed perspective about many aspects of the science of food phenolics in cereals and their processing by-products.

Phenolic acids detected in rice using LC and MS-based methods are shown in Table 1, while the contents of soluble and insoluble-bound phenolics in rice using LC and MS-based methods are presented as Supplementary Materials (Tables S1–S3). In the eighties and nineties, quantification techniques, such as gas chromatography–mass spectrometry (GC-MS), confirmed that ferulic acid was the major phenolic compound in rice and rice flour. It was also reported that this substance was primarily located in the cell wall of the plant material, covalently bound to other macromolecules (e.g., proteins, cellulose, hemicellulose, and pectin, among others). Likewise, ferulic acid has been suggested to be bound to carbohydrates (e.g., arabinose and xylose) by an ester linkage [101]. Hence, it would not be possible to extract it with mixtures of water and organic solvents such as methanol, ethanol, and acetone.

The cell wall material from wheat has been found to contain polysaccharides (66%), lignin (12%), protein (6%), ash (4%), and moisture (12%), while the respective values for rice bran were 62.4, 12, 9, 2.7, and 15% [104,105,106]. According to Whitmore [101], the phenolic–carbohydrate complex could be released by cellulase, not by pronase or a mixture of hemicellulases. Some pioneer studies from the eighties suggested alkaline hydrolysis to release phenolics from their soluble esterified quantitatively and insoluble-bound forms in wheat, rice, and other food matrices [107,108]. These studies gave support to an earlier study [99] that showed that ferulic, and sinapic acids could be bound to several sugars (e.g., fructose, mannose, and glucose), amino acids (e.g., alanine, glutamic acid, and aspartic acid), and lipids (e.g., palmitic acid). According to Klepacka and Fornal [109], ferulic acid forms chemical bonds with tyrosine and cysteine. Furthermore, esterified molecules are bonded with hemicellulose chains, mainly with arabinose residues, while binding with lignin also occurs via ether linkages. Thirty-five years after the study published by Sosulski et al. [107], an updated procedure by de Camargo et al. [97] was employed to release soluble etherified phenolics such as *p*-coumaric acid, ellagic acid, catechin, epicatechin, gallocatechin, procyanidin dimer A, procyanidin dimer B, procyanidin trimer, and procyanidin tetramer A by acidic hydrolysis. The mentioned compounds were identified and quantified by HPLC–DAD–ESI–MS^n^. Therefore, the soluble fraction could be fractionated into three sub-fractions (e.g., free, esterified, and etherified).

The subsequent decades brought many contributions to the current knowledge about phenolic compounds in wheat, rice, and their processing by-products, especially when it comes to unconventional cultivars, such as black, brown, red, and wild rice [110,111,112]. The database on flavonoids of rice, including proanthocyanidins, is shown in Table 2. Zhou et al. [110] used alkaline extraction and enzymatic hydrolysis to release insoluble bound-phenolics from three rice (*Oryza sativa* L.) cultivars (Koshihikari, medium grain; Kyeema, aromatic long grain; Doongara, long grain). The phenolic composition of brown rice (before milling) and white rice (no bran/germ) was compared. While the phenolic profile did not differ significantly between these cultivars, the samples of brown rice showed up to 5.5 times higher contents of total phenolic acids (338 versus 61 mg/kg dry grain using alkaline hydrolysis) than white rice as evaluated by HPLC, which proved that the fractions that are usually discarded after polishing show great potential as sources of natural antioxidants. Ferulic, *p*-coumaric, gallic, vanillic, caffeic, and syringic acids were identified and quantified in the tested samples. Insoluble-bound phenolics rendered a significant contribution to the TPC of the feedstocks, especially syringic (up to 97%), *p*-coumaric (up to 97%), gallic (up to 94%), and ferulic (up to 91%) acids in brown rice. In white rice, insoluble-bound ferulic (up to 94%), vanillic (up to 94%), and gallic (up to 92%) acids had the most significant impact. A similar study was carried out by Vichapong et al. [111] using six cultivars of Thai rice: Jasmine, Pathum Thani 1, Chai Nat 1, Red Jasmine, Glutinous, and Black Glutinous. Likewise, unpolished rice (0.62–1.25 mg/100 g of *p*-coumaric acid; 1.13–5.54 mg/100 g of ferulic acid) was found to be higher in insoluble-bound phenolic compounds than polished rice (0.15–0.31 mg/100 g of *p*-coumaric acid; 0.18–2.41 mg/100 g of ferulic acid). Of all rice cultivars, Black Glutinous was the one that presented the highest content of soluble and insoluble bound-phenolic acids and flavonoids.

Although available on a smaller scale, some types of pigmented rice have gained attention lately due to evidence linking their regular consumption with an array of beneficial health effects, including atherosclerosis prevention [113,114]. Furthermore, they are regarded as better sources of natural antioxidants when compared with white rice, which has been explained by their distinct phenolic composition [115]. Black rice, also known as purple rice, is characterized by intense black color and usually turns deep purple during cooking, likely due to the conversion of anthocyanins into new-colored phenolic compounds. Moreover, this cultivar has also been associated with high levels of phenolic acids and other flavonoids, making it more appealing from a functional standpoint than polished white rice. However, black rice yields are lower than white rice, making it less available and consequently more expensive. New black rice cultivars with higher yields and a desired phytochemical profile may be obtained through breeding to overcome this challenge [21].

**Table 2:** Flavonoids and proanthocyanidins detected in rice using HPLC and MS-based methods.

MF	Identity	Ion *m*/*z*	Method	SI	Ref.
C_15_H_10_O	kaempferol	285.0604	UPLC-Triple/TOF-MS	(−)	[102]
C_15_H_14_O_6_	catechin	291.0863	HPLC-LTQ-Orbitrap-MS^n^	(+)	[103]
C_15_H_14_O_6_	epicatechin	291.0863	HPLC-LTQ-Orbitrap-MS^n^	(+)	[103]
C_15_H_10_O_7_	quercetin	303.0499	HPLC-LTQ-Orbitrap-MS^n^	(+)	[103]
C_15_H_14_O_7_	epigallocatechin	307.0812	HPLC-LTQ-Orbitrap-MS^n^	(+)	[103]
C_17_H_14_O_7_	tricin	331.0812	HPLC-LTQ-Orbitrap-MS^n^	(+)	[103]
C_21_H_20_O_10_	apigenin 8-C-glucoside (vitexin)	431.0978	UHPLC-DAD-ESI-Q-TOF-MS	(−)	[116]
C_21_H_20_O_10_	apigenin 6-C-glucoside (Isovitexin)	431.0978	UHPLC-DAD-ESI-Q-TOF-MS	(−)	[116]
C_21_H_20_O_11_	luteolin 6-C-glucoside (Isoorientin)	447.0927	UHPLC-DAD-ESI-Q-TOF-MS	(−)	[116]
C_21_H_20_O_11_	luteolin 8-C-glucoside (Orientin)	447.0927	UHPLC-DAD-ESI-Q-TOF-MS	(−)	[116]
C_21_H_22_O_11_	eriodictyol 7-*O*-hexoside	451.1235	HPLC-LTQ-Orbitrap-MS^n^	(+)	[103]
C_22_H_22_O_11_	chrysoeriol 6-C-glucoside (Isoscoparin)	461.1084	UHPLC-DAD-ESI-Q-TOF-MS	(−)	[116]
C_22_H_22_O_11_	chrysoeriol 8-C-glucoside (Scoparin)	461.1084	UHPLC-DAD-ESI-Q-TOF-MS	(−)	[116]
C_25_H_26_O_13_	6,8-di-C-pentosyl apigenin	535.1446	HPLC-LTQ-Orbitrap-MS^n^	(+)	[103]
C_26_H_28_O_14_	apigenin 6-C-hexosyl-8-C-pentoside	563.1401	UHPLC-DAD-ESI-Q-TOF-MS	(−)	[116]
C_26_H_28_O_14_	apigenin 6-C-pentosyl-8-C-hexoside	563.1401	UHPLC-DAD-ESI-Q-TOF-MS	(−)	[116]
C_26_H_28_O_14_	6-C-pentosyl-8-C-hexosyl apigenin	565.1552	HPLC-LTQ-Orbitrap-MS^n^	(+)	[103]
C_26_H_28_O_15_	luteolin 6-C-pentosyl-8-C-hexoside (isomer 1)	579.1350	UHPLC-DAD-ESI-Q-TOF-MS	(−)	[116]
C_27_H_30_O_15_	apigenin 6,8-di-C-hexoside	593.1506	UHPLC-DAD-ESI-Q-TOF-MS	(−)	[116]
C_27_H_30_O_15_	vitexin 2″-*O*-glucoside	593.1506	UHPLC-DAD-ESI-Q-TOF-MS	(−)	[116]
C_27_H_30_O_15_	6,8-di-C-hexosyl apigenin	595.1657	HPLC-LTQ-Orbitrap-MSn	(+)	[103]
C_27_H_30_O_16_	isoorientin 2″-*O*-glucoside	609.1453	UHPLC-DAD-ESI-Q-TOF-MS	(−)	[116]
C_27_H_30_O_16_	luteolin 6,8-di-C-hexoside	609.1456	UHPLC-DAD-ESI-Q-TOF-MS	(−)	[116]
C_27_H_30_O_16_	rutin	611.1607	HPLC-LTQ-Orbitrap-MS^n^	(+)	[103]
C_28_H_32_O_16_	chrysoeriol 6,8-di-C-hexoside	623.1611	UHPLC-DAD-ESI-Q-TOF-MS	(−)	[116]
C_28_H_32_O_16_	chrysoeriol 6″-*O*-hexosyl-6-C-hexoside	623.1612	UHPLC-DAD-ESI-Q-TOF-MS	(−)	[116]
C_30_H_26_O_12_	A-type procyanidin dimer	577.1341	HPLC-LTQ-Orbitrap-MS^n^	(+)	[103]
C_30_H_26_O_12_	Procyanidin B1	579.1497	HPLC-LTQ-Orbitrap-MS^n^	(+)	[103]
C_30_H_26_O_12_	Procyanidin B2	579.1497	HPLC-LTQ-Orbitrap-MS^n^	(+)	[103]
C_30_H_26_O_12_	Procyanidin B3	579.1497	HPLC-LTQ-Orbitrap-MS^n^	(+)	[103]
C_45_H_34_O_18_	A-type procyanidin trimer	863.1818	HPLC-LTQ-Orbitrap-MS^n^	(+)	[103]
C_45_H_36_O_18_	A-type procyanidin trimer	865.1974	HPLC-LTQ-Orbitrap-MS^n^	(+)	[103]
C_45_H_38_O_18_	Procyanidin C1	867.2131	HPLC-LTQ-Orbitrap-MS^n^	(+)	[103]
C_60_H_48_O_24_	A-type procyanidin tetramer	1153.2608	HPLC-LTQ-Orbitrap-MS^n^	(+)	[103]
C_60_H_50_O_24_	B-type procyanidin tetramer	1155.2765	HPLC-LTQ-Orbitrap-MS^n^	(+)	[103]

Molecular formula; SI, selected ion; (−), negative mode; (+), positive mode.

Zhang et al. [21] evaluated the phenolic composition of 15 hybrid rice samples that crossed white and black cultivars. The hybrids were grouped based on their colors: five white, five light-purple, and five black. In terms of TPC, light-purple and black cultivars outperformed the white ones, with YF53 (black) presenting the highest content of soluble phenolics (16.74 mg ferulic acid equivalent (FAE)/g rice), while YF67 (light-purple) was the richest sample when it comes to insoluble-bound phenolics (4.79 mg FAE/g rice). Ferulic acid represented around 65% of all phenolic compounds in the insoluble-bound fraction. As expected, black cultivars were the highest in total soluble anthocyanins (1.47–2.07 mg cyanidin-3-glucoside equivalent/g), with cyanidin-3-glucoside and peonidin-3-glucoside representing the major compounds from this group. This amount was eight times higher than those found in light-purple grains.

Another study [40] has found that during the grain’s developmental stages, the TPC of black rice increased, especially regarding soluble and insoluble-bound phenolics. On the other hand, white and red rice had higher phenolic content during the first week of development. Once they reached the fully matured stage, the TPC decreased to values as low as 82% less than their initial TPC. Total soluble anthocyanin content decreased slightly in black rice after the grain fully matured. The peak in terms of anthocyanin was reached between the second and third weeks (174.71 and 172.21 mg cyanidin-3-glucoside equivalent/100 g rice grain, respectively).

Amongst all by-products of black rice evaluated by LC-MS/MS [40,117], the bran is the best source of soluble anthocyanins, with 3.576 mg of cyanidin 3-O-glucoside/g, as opposed to 0.116 mg/g quantified in the embryo and 0.515 mg/g in whole grain. The endosperm did not contain anthocyanins. Black rice’s bran also carried the highest content of soluble (free/conjugated) phenolic acids, such as protocatechuic acid (34.21 mg/100 g), vanillic acid (31.67 mg/100 g), and sinapic acid (7.27 mg/100 g). Interestingly, the embryo portion also demonstrated to be a rich source of soluble phenolics, namely, ferulic acid (53.24 mg/100 g, 89% higher than the amount quantified in the bran), syringic acid (5.54 mg/100 g, not detected in the bran), *p*-coumaric acid (5.26 mg/100 g, 23% higher than bran), and isoferulic acid (12.92 mg/100 g, 70% higher than bran). The sum of the insoluble-bound phenolic acids quantified by LC-MS/MS accounted for 166.88 mg/100 g (protocatechuic, vanillic, syringic, *p*-coumaric, ferulic sinapic, and isoferulic acids) in the bran. The embryo presented 83.99 mg/100 g (vanillic, syringic, *p*-coumaric, ferulic, sinapic, and isoferulic), representing 49% less phenolic content than the bran.

Similar to the black cultivar, red rice is also rich in phenolic acids and anthocyanins, surpassing the nutritional value of polished white rice. Shen et al. [118] reported that the total soluble phenolic content of five cultivars of red rice from China ranged between 165.8 and 731.8 mg GAE/g, while the white one was 108.1–251.4 mg GAE/g. These authors also analyzed the content of flavonoids present in the soluble fraction. As expected, red rice showed a higher amount (108.7–190.3 mg rutin equivalent (RE)/100 g) compared with the white one (88.6–170.7 mg RE/100 g). However, all samples showed black rice with the highest flavonoid content (187.6–286.3 mg RE/100 g), which can probably be related to the presence of anthocyanins. According to Laokuldilok et al. [119], the amount of soluble anthocyanins present in the bran of red rice represents only 10% of what was found in black rice’s bran. However, since most of the studies focused on the phenolic acids, the literature about red rice is still limited in what concerns the identification and quantification of flavonoids present, including anthocyanins.

Another type of rice rich in phenolic compounds is wild rice (*Zizaniae palustris* and *Zizaniae aquatica*). This cultivar originated from the northern United States and southern Canada, where the indigenous population consumed it. Wild rice is commercially produced and used in various specialty foods due to its unique flavor and appearance. Wild rice is consumed as a whole grain (dehulled), preserving the bran and the germ, which, regardless of species and cultivar, are the most important fractions in phenolic compounds [120]. According to Qiu et al. [121], the content of soluble phenolics in raw wild rice (2472–4072 mg/kg) was 10–15 times higher than that of white rice (279 mg/kg). These authors also demonstrated that quick-cooking of wild rice, which involves soaking, cooking, and drying, significantly lowers the TPC (around 50% of loss). This effect was attributed to the leaching of soluble phenolic compounds upon processing and the destruction and transformation of chemical structures present in the raw grain.

The primary soluble phenolic acids reported for wild rice are ferulic (150.8–234.9 mg/kg), gallic (64.0–154.0 mg/kg), and sinapic (25.4–53.6 mg/kg) acids. However, protocatechuic, ellagic, vanillic, and *p*-coumaric acids were also detected in lower amounts. For soluble flavonoids, rutin (73.4–128.7 mg/kg), epicatechin (37.5–78.3 mg/kg), epigallocatechin (8.7–27.7 mg/kg), and catechin (2.1–22.1) were the major compounds [122]. Another study [121] also reported the presence of catechin, epicatechin, and their oligomeric procyanidins (e.g., dimer, trimer, tetramer, and pentamers) in wild rice after the fractionation of the crude phenolic extract.

The concentrations of procyanidin monomers (7.16–18.15 μg/g), dimers (12.42–42.42 μg/g), trimers (tr-69.18 μg/g), tetramers (nd-52.67 μg/g), and pentamers (nd-61.62) refer to the soluble fraction of raw wild rice. As evaluated by HPLC-MS/MS, the total procyanidins levels ranged from 7.16 to 239.22 μg/g. It is important to mention that procyanidins were not found in white rice. The presence of catechin (15.6–21.3 μg/g), epicatechin (24.3–43.3 μg/g), procyanidin B (110.2–13.0 μg/g), procyanidin B2 (5.0–5.5 μg/g), procyanidin B3 (6.3–9.4 μg/g), and procyanidin C1 (17.0–24.2 μg/g) in wild rice was reported by Chu et al. [103]. The corresponding concentrations of catechin, epicatechin, procyanidin B1, procyanidin B2, procyanidin B3, and procyanidin C1 in red rice were 6.6, 3.5, 7.0, 2.4, 3.4, 6.0, all of them being lower than that of wild rice. Furthermore, lending support to the study by Qiu et al. [121], none of the procyanidins were detected in white rice [103].

Besides flavan-3-ols, these authors also identified flavone glycosides (e.g., diglucosyl apigenin, glucosyl-arabinosyl apigenin, and diarabinosyl apigenin). In the insoluble-bound fraction of wild rice [122], ferulic acid was also the most abundant compound (100.3–145.1 mg/kg), followed by sinapic acid (22.2–45.1 mg/kg). Chlorogenic acid was detected in trace amounts in the soluble fraction and was significant in the insoluble-bound form (8.7–19.2 mg/kg). Ellagic acid was also seen in a greater amount in this fraction (11.2–81.4 mg/kg). Flavonoids were significantly higher in the insoluble-bound fraction, with rutin (116.5–240.7 mg/kg) and epigallocatechin (21.3–51.6 mg/kg) as the major compounds. Catechin, epicatechin, quercetin, and kaempferol were also detected in lower proportions. According to the authors [122], the region where the grains were grown and the climatic and harvest conditions affected the phenolic composition in wild rice.

Regardless of the rice cultivar, its hull is generally removed before human consumption. Therefore, being considered a residue with little economic value. However, like bran, this fraction still contains phenolic antioxidants, mainly responsible for protecting the intact seed against biotic, abiotic, and oxidative stress [20]. If recovered, this by-product can be a valuable source for many applications, including its use as a functional food ingredient and potential manufacture of nutraceuticals and food supplements [18,39,123].

The hull removed during white rice processing was the subject of a study by Butsat et al. [39]. These authors evaluated its phytochemical composition during the different stages of grain development. The TPC for soluble phenolics (1.1–2.1 mg GAE/g) decreased by almost 50% throughout the developmental stages, which was not observed for the insoluble-bound fraction (6.6–8.0 mg GAE/g), where the TPC showed only slight variations depending on the growth stage, a phenomenon also reported by Shao et al. [40] for white, red, and black rice grains. The phenolic acid composition of the hull was similar to the other parts of the grain, with *p*-coumaric (soluble = 14.8–32.5 μg/g, bound = 5057–8027 μg/g), ferulic (soluble = 18.1–64.2 μg/g, bound = 1825–1983 μg/g), and syringic (soluble = 2.6–12.1 μg/g, bound = 247.2–310.5 μg/g) acids as the dominant compounds. Gallic, protocatechuic, and chlorogenic acids were detected only in the fraction containing soluble phenolics.

Interestingly, unlike the other parts of the rice grain, ferulic acid is not the most abundant phenolic in the hull [124,125]. These authors reported *p*-coumaric acid as the primary phenolic acid in rice hull, while this fraction showed myricetin (23.62–32.62 μg/g of dry weight) as its main flavonoid [125]. However, as discussed here, it is important to remember that the phytochemical profile may vary depending on the rice cultivar.

Jha et al. [126] investigated the soluble phenolic composition of black rice hull and reported quinic acid as the primary phenolic acid (2682.37 mg/kg), followed by chlorogenic acid (123.65 mg/kg), syringic acid (65.34 mg/kg), *p*-coumaric acid (31.19 mg/kg), and ferulic acid (20.45 mg/kg). As for the flavonoid composition, quercetin was the primary compound, with 46.84 mg/kg, followed by smaller amounts of catechin (1.72 mg/kg) and apigenin (1.34 mg/kg). However, phytochemicals in rice hull from colored cultivars are a relatively underexplored topic. Although extensive work has been performed on the phenolic composition of pigmented rice bran and whole grain [21,40,80], the hull fraction has been less explored [39,127]. More studies are needed to elucidate the phytochemical profile of this rice fraction, especially by employing fractionation techniques to release soluble compounds from their esterified and etherified forms and to hydrolyze and further identify and quantify those liberated from the cell wall of the plant material, also known as insoluble-bound.

In classical studies [128], the antioxidant properties of wheat have been mainly attributed to monomeric phenolics (e.g., vanillic, *p*-coumaric, ferulic, and sinapic acids). However, more recently, the use of mass spectrometry has allowed the identification of molecules bearing a higher structural complexity, such as dimers and trimers of ferulic acids, also known as diferulic and triferulic acids, respectively [129,130], as well as flavonoids such as apigenin, luteolin, and chrysoeriol and their derivatives [131,132,133]. Soluble phenolics from wheat are extracted mainly with aqueous mixtures of ethanol or methanol [130,131,133,134]. However, a recent study [98] has demonstrated that aqueous acetone (1:1, *v*/*v*) is a better choice than methanolic extraction. Furthermore, the insoluble-bound fraction generally recovers after alkaline hydrolysis [42,135]. The concentrations of soluble and insoluble-bound phenolics in wheat (using LC and MS-based methods) are presented as Supplementary Materials (Tables S4 and S5).

The environmental and genotype effects on phenolics from several wheat cultivars, including common wheat, have been addressed by di Silvestro et al. [134]. According to these authors, the red grain cultivars Andriolo, Gentil rosso, and Verna were considered the most promising for breeding new cultivars bearing a high nutraceutical value. Likewise, the phenolic profile of ancient and modern durum wheat genotypes was addressed by di Loreto et al. [136]. Other groups have also conducted comparative studies [112,118], and old durum wheat cultivars have been suggested to offer unique phytochemical profiles that could render better health effects than those of modern durum wheat [131]. However, in terms of TPC, Tunisian durum wheat was ranked differently (modern > landraces > old) by Boukid et al. [130]. In addition to ferulic acid (which is reported by most studies) (Table 3), these authors also provided the concentrations of other phenolics such as diferulic (three isomers) and triferulic acids in six landraces, as well as in three old and six modern genotypes, which was possible due to use of UHPLC/MS^n^ analyses.

Significant phenolic profile differences between modern and ancient genotypes of common wheat have also been reported [140]. Anthocyanins have been identified in the bran of dark-blue-grained wheat (Triticum aestivum L. cv. Hedong Wumai), with cyanidin-3-glucoside making the highest contribution; although, cyanidin-3-galactoside, pelargornidin-3-glucoside, and peonidin-3-glucoside were also present [141]. According to Montevecchi et al. [142], the high rusticity and adaptability to marginal soils and the richness in (poly)phenols of ancient wheat cultivars could facilitate their use in organic or sustainable agriculture. Beyond phenolic acids, a myriad of flavonoids and derivatives thereof have been detected by various HPLC and MS-based methods (Table 4).

The study by Irakli et al. [143] demonstrates that the concentration of soluble and insoluble-bound gallic acid, soluble and insoluble-bound 4-hydroxybenzoic acid, insoluble-bound vanillic acid, soluble caffeic acid, soluble *p*-coumaric acid, soluble ferulic acid between durum and bread wheat may be negligible (Supplementary Material, Tables S4 and S5). The same trend was found for the total soluble phenolic acid content (6.32–7.30 μg/g) as evaluated by HPLC-DAD. As for the insoluble-bound fraction, bread wheat (335.26 μg/g) showed slightly higher contents of total phenolic acids than durum wheat (291.32 μg/g) using the same method.

The soluble anthocyanin content in purple wheat was addressed by Hosseinian [144]. According to these authors, cyanidin 3-glucoside (103.0 mg/kg), cyanidin 3-galactoside (72.0 mg/kg), and malvidin 3-glucoside (51.6 mg/kg) were the main anthocyanins found. The presence of procyanidin B in the soluble and insoluble-bound fraction of wheat has been reported by studies [131,132,138]. However, in contrast to what has been found for rice, the concentrations remain to be determined.

Following the same trend of other cereal products, refined wheat flour exhibits a much lower concentration of insoluble-bound ferulic acid (12–27-fold) than whole wheat flour. Likewise, the bread produced with refined flour contained just a small percentage (5–12%) of the same compound compared to the content of bread made with whole wheat flour (231–393 µg ferulic acid/g of the dry sample) as evaluated by HPLC and confirmed by LC-MS/MS [135]. The same trend was reported by Mattila et al. [145]. According to these authors, the total phenolic acids content in white wheat flour is about 150–167 mg/kg fresh weight, much lower than that in whole grain (i.e., 1342 mg/kg fresh weight). The phenolic profile of soluble and insoluble-bound phenolics during the production of bread from purple wheat grains was addressed by Yu and Beta [96]. Collected bread crust and crumb samples collected after 30 min fermenting, 65 min fermenting, and baking were examined. These authors showed that bread-making significantly compromised the anthocyanin content of purple wheat bread, which did not occur with phenolic acids. The retention of anthocyanins range was quite similar in bread loaf made with Indigo (44.7%) and Konini wheat flour (46.3%). In contrast, fermentation improved soluble phenolics’ content since ferulic acid’s concentration increased from 2.02–2.50 (flour) to 13.25–15.01 μg/g of dry weight (65 min of fermentation). Additionally, soluble *p*-hydroxybenzoic acid, which was absent in the flour, was present in the bread loaf, thus suggesting their release from soluble-esters and insoluble-bound forms.

It is important to note that storage conditions may also impact the phenolic composition of wheat and rice. Zhou et al. [146] submitted brown rice samples to simulated storage conditions (4 °C and 37 °C) for six months, then analyzed the effects on the content of phenolic acids, flavonoids, and proanthocyanidins. TPC (soluble and insoluble-bound fractions) showed a slight increase after two months of storage at both temperatures but significantly decreased for samples at 37 °C after this period (15–20% decrease). The authors hypothesized that phenolics might be involved in chemical reactions occurring during rice aging, which led to their decline during storage. The same trend was observed for total flavonoids, total proanthocyanidins, and ferulic acid contents. Although the presence of proanthocyanidins in rice has been little explored, some authors have detected their presence in this cereal by HPLC and MS-based methods (Table 4). A similar result was obtained by Lang et al. [147] when black rice samples were stored at 25 °C for 12 months. Under a conventional atmosphere, TPC decreased in the soluble and insoluble-bound fractions during the storage period, but this was prevented in samples stored under a nitrogen atmosphere. The content of soluble flavonoids increased during storage under a conventional atmosphere, which was attributed to the hydrolysis of insoluble-bound flavonoids by enzymatic action. Moreover, after 12 months of storage, the sum of individual phenolics decreased by 50%, with only caffeic acid maintaining the same concentration before and after storage. The potential phenolic acids and other polyphenols bioactivities of rice and wheat are discussed in subsequent sections.

## 5. Bioefficiency (Bioaccessibility and Bioavailability) of Phenolics from Rice, Wheat, and By-Products Thereof

The term bioavailability has been defined as the percentage or fraction of a dose of a compound (natural or not) that, when given orally, may be recovered systemically in plasma or blood within a lapse of time that is defined by the area under the curve AUC (plasma/blood concentration along time, until its approximate disappearance). Furthermore, bioaccessibility has been described as the digestion and absorption efficiency of food, a natural compound, or a drug orally administered, which can be expressed as the percentage of the total amount released and absorbed. Bioaccessibility falls within the scope of bioavailability and accounts for the amount of a substance that is assimilated and used for storage and metabolic function. It has been suggested that both concepts are similar. Hence, they could be grouped in a broader definition: bioefficiency [148,149], which the scientific community may also use.

Many factors influence the bioefficiency of phenolic compounds, namely, their structural characteristics, interactions with other components of the food matrix, and the health condition of the host. Therefore, being present in large amounts does not necessarily mean that a particular phenolic will exist in a physiologically significant concentration upon gastrointestinal digestion and, more specifically, under systemic conditions. That is why chemical and cell-based assays, although able to provide valuable information on the potential action of phenolics, may not reflect what takes place after all steps that follow the consumption of phenolic-rich feedstocks [18,148].

Prooxidant compounds (e.g., nonsteroidal anti-inflammatory drugs, mycotoxins, aldehydes, copper and iron ions, haem proteins, dietary nitrite and sulfite, myoglobin, and caffeine) are found in the gastrointestinal tract [150,151,152,153,154,155,156]. Kanner et al. [157] incubated heated muscle tissue in simulated gastric fluid. They reported an enhancement of accumulation of hydroperoxides, which dropped to zero in the presence of polyphenols, thus demonstrating that phenolics from the starting material may be at least partially utilized before any absorption.

Several of the cells present in the gastrointestinal tract have an important role in the immune system. Food bacteria may induce gastrointestinal inflammation, which may be counteracted by phenolic compounds [158,159]. In addition, as multifunctional compounds, polyphenols may also inhibit digestive enzymes (e.g., α-amylase, α-glucosidase, and lipase) by forming complexes with these enzymes via hydrogen bonds or the addition of nucleophiles to oxidized quinones [20,160,161]. Therefore, due to their gastrointestinal consumption as an antioxidant, anti-inflammatory, and interaction or inhibition of digestive enzymes, phenolics initially present in the starting material or released during digestion may not be able to enter the bloodstream and organs in the human body.

Zhao et al. [162] evaluated the digestion and absorption of ferulic acids and their respective sugar esters (5-O-feruloyl-L-arabinofuranose and feruloyl-arabinoxylan) in rats. According to these authors, free ferulic acid as such or as a part of simple ferulic acid sugar esters (e.g., 5-O-feruloyl-L-arabinofuranose) could be absorbed directly in the upper part of the gut, while feruloylated polysaccharides together with the part of simple ferulic acid sugar esters that are not absorbed in the foregut reach the caecum.

Kern et al. [163] conducted a human trial addressing the absorption of hydroxycinnamates after high-bran cereal consumption. These authors concluded that the major hydroxycinnamic acids taken up after eating a high-bran cereal were ferulic acid and sinapic acid, with nanomolar levels detected in the plasma. They highlighted that the absorption took place mainly in the small intestine. In addition, they suggested that covalently bound diferulic acids are either not absorbed or absorbed only in minimal amounts.

Using Caco-2 cells, Konish et al. [164] suggested that ferulic acid could be absorbed via monocarboxylic acid transporters (MCTs). The transport of functional food extracts, including phenolic compounds, has recently been revisited by Iftikhar et al. [165]. In rats administered 2.25 µmol of ferulic acid and other phenolic acids, Konish et al. [166] showed that the plasma concentration of ferulic acid peaked 5 min after administration in the stomach of the animals. The concentrations were in the increasing order of gallic = chlorogenic < caffeic < and *p*-coumaric = ferulic acid. Similar to intestinal absorption, the authors suggested that MCT could be involved in the gastric absorption of phenolic acids. Furthermore, the mentioned increasing order in the gastric absorption efficiency correlated with their individual affinity for MCT in Caco-2 cells, as investigated by the same research team [167,168,169]. Therefore, supporting the hypothesis that the MCT-mediated absorption system could be involved not only in the intestinal but also in the gastric absorption of phenolic acids in vivo.

Zhao et al. [170] proposed the metabolic fate of free ferulic acid in rat stomach. Ferulic acid is absorbed in the free form by the gastric mucosa, and then it is transported through the portal vein, entering the liver and conjugating to produce glucuronide/sulfate. The remaining free ferulic acid and its conjugated forms enter the circulatory system and are distributed to peripheral tissues. After gastrointestinal digestion, some soluble free phenolics are readily absorbed in the small intestine, where they undergo a series of conjugation by methylation, sulfation, glucuronidation, or a combination of them, being further introduced in the blood circulation system, traveling through the bloodstream until reaching different organs or being excreted in the urine. Some enzymes, such as sulfotransferases, uridine-5′-diphosphate glucuronosyltransferases, and catechol-O-methyltransferases, are involved in the respective production of glucuronidated, sulfated, and methylated conjugates during phase II metabolism, respectively. Otherwise, the metabolites may efflux back into the lumen of the small intestine [171].

The hydroxycinnamic acids (especially ferulic and *p*-coumaric acids abundant in rice, wheat, and by-products) are in the insoluble-bound form, primarily bound through ester, ether, and carbon–carbon bonds in the cell wall matrix to insoluble macromolecules such as cellulose, pectin, and structural proteins [172]. Therefore, insoluble-bound phenolics are not absorbed in the small intestine, moving directly to the colon. The colon is colonized by various microorganisms, such as Bifidobacterium spp. and Lactobacillus spp. These microorganisms can hydrolyze the covalent bonds that link the phenolics to the cell matrix because they secrete extracellular enzymes such as carbohydrases and proteases.

As for phenolic acid esters, there are no esterases in the human tissues capable of hydrolyzing ester bonds, which leads them to the colonic fermentation pathway. The phenolic compounds undergoing this metabolic route account for approximately 90–95% of all polyphenols consumed in the diet, which explains the low bioavailability demonstrated by some phenolic classes, such as flavonoids, in animal and in in vitro studies [172]. Besides insoluble-bound phenolics, metabolized and excreted polyphenols can also reach the colon as glucuronides. With approximately 1012 microorganisms/cm3, the colon possesses great catalytic and hydrolytic activity, which leads to rapid deconjugation reactions. Complex polyphenols are broken down into simpler compounds in the colonic environment, such as phenolic acids. These simpler compounds in the colon are associated with an increased accumulation of short-chain fatty acids, which decrease the pH in the colon’s lumen and reduce the growth of harmful microorganisms [17,148,172,173].

Lending support to the importance of phenolic acids linked to the insoluble-bound fraction, Rondini et al. [174] showed that the presence of ferulic acid–arabinoxylans bonds in the food matrix (wheat bran) increases the time ferulic acid is retained in the organism (i.e., free + conjugated, after in vivo metabolization) rather than being excreted in the urine after 24 h of the intake. While the contents of these compounds decreased to zero after 4.5 h in the plasma of rats that ingested free ferulic acid, the concentration of the same compounds remained unchanged for 24 h in the plasma of rats that received wheat bran as a source of insoluble-bound ferulic acid. Therefore, whole cereals may be considered a good choice regarding the slow release of phenolic acids into the bloodstream.

An in vivo study [175] showed that rats fed a ferulic acid-enriched diet (50 µmol FA/day) with lower cereal intake resulted in around 50% of its absorption, expressed as the percentage of the ingested dose recovered in urine. The plasma concentration was 1 µmol/L, significantly higher than that (0.2–0.3 µmol/L) found in rats fed a high cereal-based diet containing 56–81 µmol FA/day. According to the authors, the association of ferulic acid with fiber limits its bioavailability. Human intervention studies confirmed that soluble ferulic acid is readily absorbed in the small intestine, while its bound form undergoes colonic fermentation [163]. Some flavonoids are also reported as having low bioavailability, namely, proanthocyanidins (average of 0.2% of the intake detected in urinary excretion), galloylated tea catechins (not recovered in urine), and anthocyanins (0.5%) [18,176].

Janarny and Gunathilake [177] evaluated the solid-state fermentation by *Rhizopus oryzae* to see if it could enhance the bioefficiency of phenolic compounds from rice bran. Interestingly, red rice cultivars presented higher total anthocyanin content in unfermented samples (2.97 and 9.55 mg cyanidin3-glucoside/g FW) than their fermented counterparts (0.01 and 0.21 mg Cy 3-glc/g), demonstrating the opposite of what was observed for white rice bran. The samples were submitted to in vitro gastrointestinal digestion with a total anthocyanin recovery of 22.42–22.73% for unfermented white rice bran and 14.21–27.64% for unfermented red rice bran in the gastric phase. However, anthocyanin recovery decreased significantly after unfermented samples had undergone the intestinal phase (0.07–12.46%), with the highest recovery recorded for a white rice bran cultivar, while the lowest yield was reported for a red rice bran cultivar. Meanwhile, fermentation significantly increased the bioefficiency of rice bran anthocyanins in both gastric (25.40–66.39%) and intestinal (9.79–43.39%) phases. According to these authors, this could be primarily due to the release of extracellular enzymes by fungal metabolism, which would be responsible for cleaving bound phenolics, consequently enhancing their bioaccessibility.

Spray-drying-based microencapsulation has been used by [178] as a strategy to increase the release of anthocyanins from a Thai black rice cultivar. An anthocyanin-rich fraction extracted from this raw material was compared with microcapsules containing 25 mg of anthocyanins and using combinations of maltodextrin (M), gum Arabic (G), and whey protein isolate (W). The in vitro gastrointestinal digestion showed that in the gastric phase, the non-encapsulated extract had released around 100% of its anthocyanin content after 30 min, with M, MG (7:3), and MW (7:3) presenting the same release rate. However, all samples showed a decrease in their anthocyanin release upon the intestinal phase, where the encapsulated samples outperformed the extract. In this phase, MW (7:3) microcapsule released the highest level of anthocyanins from all samples (around 60–70% between 3 and 6 h of intestinal digestion), whereas the extract showed release rates of less than 40%. Therefore, this study demonstrated that the microencapsulation technique employed provided an efficient tool to enhance the bioaccessibility of anthocyanins from black rice.

Flavonoids are present in the food matrix as aglycones or glycosides, as seen in the case of apigenin, which is widely available in wheat [179]. Flavonoid glycoside metabolism usually follows two main routes: going to the large intestine to be fermented into aglycone by the colonic bacteria or absorbed in the small intestine and liver. Compounds undergoing the latter route are metabolized by phase II enzymes, promoting their deglycosylation, resulting in derivatives that may undergo hydroxylation, methylation, and reduction in the liver. On the other hand, the released aglycones may be sulfated or glucuronidated, becoming flavonoid metabolites. The deglycosylation step favors the absorption of aglycones, which are more hydrophobic than their glycoside counterparts. Hence, they can cross the membrane of epithelial cells by passive diffusion [180].

The metabolic difference between flavonoid aglycones and glycosides raises the question of which of those two forms renders the highest bioefficiency. Some authors have addressed this issue, such as [181], who studied the bioavailability of apigenin aglycone and C-glycoside (vixetin-2-O-oxyloside—VOX) using a rat model with apigenin administration into the caecum. According to the authors, apigenin glycosides can appear as O-glycosides or C-glycosides, with little information available on the metabolism of the latter group. O-Glycosides reach the small intestine and become a substrate for lactase phlorizin hydrolase, an enzyme present in the brush border, as well as intracellular β-glucosidases, with further release of their aglycones. After digestion, the authors detected apigenin aglycone and its glucuronide form in the portal blood. Meanwhile, VOX was a reduced monoglycoside, which glucuronidation transformed. Unaltered VOX was enteropathic recirculated to the gut for reabsorption from the ileum in the liver.

Food processing may help enhance the bioefficiency of cereal grain phenolics. Wang et al. [28] stated that the accessibility of insoluble-bound phenolic compounds might increase through particle size reduction, structural breakdown of cereal grains, and liberation of phenolics from their matrices using suitable extraction processes. Thermal treatment, however, could decrease their bioefficiency in case high temperatures are applied, which leads to the degradation of some phenolic compounds, such as ferulic acid, protocatechuic acid, *p*-coumaric acid, and quercetin [147]. Therefore, all those factors and processing technologies should be appropriately balanced.

The absorption and metabolism aspects of phenolic compounds present in rice, wheat, and their by-products remain unclarified. However, some interesting alternatives to increase the bioefficiency of such phenolics are starting to draw attention. Perez-Ternero et al. [182] used an enzymatic treatment with endoprotease to release ferulic acid from its insoluble-bound state in rice bran. The authors increased ferulic acid’s absorption from 0.4–5% to 18.8% in an animal trial using mice.

## 6. Antioxidant Capacity of Rice, Wheat, and By-Products Thereof

Several chemical-based antioxidant assays are widely used for screening experiments in prospecting for common and novel antioxidant sources. The study by de Camargo et al. [183] suggested that colorimetric and fluorometric assays (e.g., FRAP and ORAC) can foresee the biological activity of phenolic-rich extracts containing phenolic acids and flavonoids. The results from these chemical-based assays were further confirmed by the reduction in the activation of the nuclear factor (NF-κB), a biomarker related to the inflammatory responses mediated by oxidative stress.

Assays involving peroxyl and hydroxyl species, ferric-reducing power, and metal chelation capacity have proven to be, at least partially, biologically relevant. In contrast, DPPH and ABTS radical cations are more limited because none exist in the human body or food systems. Accordingly, there is a consensus that the results obtained from these assays may not be entirely helpful in extrapolating or predicting the fate of phenolic antioxidants in the human body [183]. However, Falcão et al. [184] also demonstrated that samples exhibiting higher antiradical activity towards DPPH radical and ABTS radical cations also show higher biological activity by reducing the activation of NF-κB using RAW 264.7 macrophages. De Camargo et al. [183] encouraged the scientific community to determine bioactive compounds by employing at least some in vitro biological methods (e.g., cell lines) and also contemplating simulated digestion; although, in vivo assessment has been regarded as being most preferred. The general aspects and the pros and cons of antioxidant methods have already been addressed [185,186,187]. Nevertheless, one should always bear in mind the primary purpose of each study.

As for agronomic and post-harvest research (e.g., storage and shelf-life studies), it is not unusual to find studies that evaluate a large number of samples [146,188], thus making the assessment in cell lines and animal models as well as human clinical trials prohibitive. Therefore, considering the literature on the potential ability of colorimetric methods in anticipating biological properties [183,184], it is possible to suggest that DPPH and ABTS, as well as FRAP and ORAC, among others (e.g., scavenging of hydroxyl radicals and metal chelation), still have room as screening methods.

Cheng et al. [189] investigated the influence of wheat form (grain, bran, and bran flour) and storage temperature (25, 60, and 100 °C) on the phenolic acid composition and antioxidant activity of the feedstock. All samples were stored for nine days, and during this period, grain samples were detected as the most stable ones in terms of antioxidant activity measured by ORAC, DPPH, ABTS, and scavenging activity of superoxide radicals generated from xanthine oxidase system. Meanwhile, bran flour presented a significant reduction (13.6–60%) in the antioxidant activity from day 2 to 5, with the samples under 100 °C most affected. According to the authors, the reduced particle size of the grain flour led to a higher exposure of the antioxidant compounds to environmental conditions, promoting their degradation.

The hydroxyl group(s) on aromatic rings provides a potential antioxidant effect, which can be explained by donating electrons or transferring hydrogen atoms to stabilize free radicals. Mechanisms by which this may take place include free-radical scavenging, action as a reducing agent, and quenching of singlet oxygen, possibly chelation of prooxidant metal ions or a combination of two or more mechanisms, depending on the chemical nature of a compound, its interactions with other substances, and the transformations that it may undergo during food processing [187,190].

**Figure 2:**
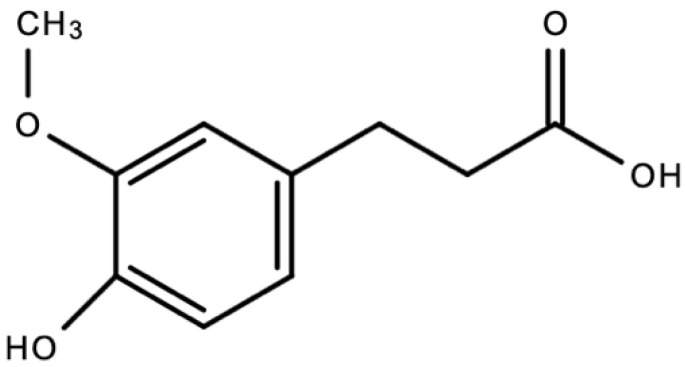
Ferulic acid structure.

Ferulic acid, the predominant phenolic acid in most rice and wheat cultivars and most of their by-products [136,191,192], carries three structural characteristics that may contribute to its alleged free radical scavenging capacity. The molecule (Figure 2) has electron-donating groups on its benzene ring, which can be transferred to excited radicals, terminating chain reactions. Ferulic acid also possesses a carboxylic group with an adjacent unsaturated C-C double bond, providing additional attack sites for free radicals, thus preventing them from reacting with other molecules, such as lipids and DNA.

According to Chen et al. [193], the number of hydroxyl groups on the phenoxyl ring of phenolic acids significantly affects their antioxidant activity, which goes up consistently when less than four OH groups are present in the structure. Due to their electron donor ability, hydroxyl groups can enhance the antioxidant power of other phenolic hydroxyls. Besides the methylation and hydroxylation pattern, a study [194] on the free radical scavenging capacity of gallic acid derivatives has demonstrated that an increase in the acyl chain length negatively affects the antioxidant power of these compounds due to steric hindrance. In addition, gallic acid derivatives with higher hydrophobicity index performed better at mitigating oxidative stress in cell lines than their hydrophilic counterparts because they can easily enter the cytoplasm and offset the formation and accumulation of reactive oxygen species.

The protective effect of ferulic and other phenolic acids towards H_2_O_2_ + UV-induced supercoiled circular DNA damage is shown in Table 5 The carboxylic group also functions as a site for bonding between ferulic acid and the cell membrane’s lipid bilayer, which enhances the protection against lipid peroxidation. This last feature is especially appreciated under physiological conditions as it may reduce oxidative stress and its health-related outcomes [195].

**Figure 3:**
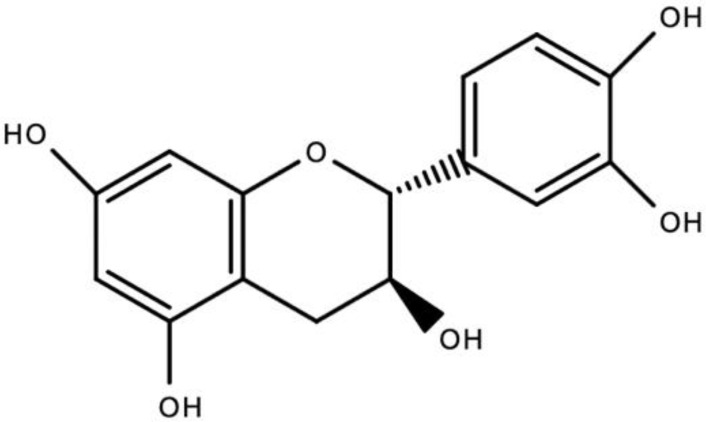
Catechin structure.

Catechin, a flavonol reported as one of the major flavonoids in rice hull [124], presents a dihydroxylation in the B ring’s 3′ and 4′ positions (Figure 3). This characteristic renders catechin the ability to act as an antioxidant by donating hydrogen atoms to free radicals, among other possible mechanisms, such as singlet oxygen quenching [196]. The relationship between the structure of phenolic compounds present in rice, wheat, and other cereal grains and their potential activity is essential for understanding the antioxidant properties of these feedstocks. Nevertheless, recent findings indicate that free radical scavenging may not be explained by a single compound but rather by the synergistic effect between all phenolic compounds present in a complex mixture and, possibility, other bioactive molecules such as carotenoids, and Maillard reaction products, among others [197,198,199]. As these compounds are not phenolic in nature, their detailed discussion falls outside the scope of this review.

Braughler et al. [200] studied how lipid peroxidation initiation is influenced by the ratio of ferric to ferrous ions. Rapid initiation of lipid peroxidation was reported when the Fe^3+^/Fe^2+^ ratios were 1:1 to 7:1. The formation of hydroxyl radicals was not detected, even though the authors did not rule out the involvement of such species in the oxidative process. Therefore, the ferric-reducing power presented by phenolic compounds helps decrease the ferric concentration. At the same time, ferrous ions initiate lipid oxidation and participate in the Fenton Reaction, which generates hydroxyl radicals, highly reactive substances that induce DNA damage. Therefore, along with reducing power, metal chelation (Table 6) has been proposed as a crucial feature in designing an “ideal antioxidant” [20,159].

The iron-chelation properties of seven phenolic acids bearing catechol and galloyl groups (e.g., caffeic, chlorogenic, gallic, protocatechuic, vanilic, syringic, and ferulic acids) were investigated by Andjelković et al. [201]. Their results showed that not all tested molecules showed complex formation (i.e., those lacking catechol or galloyl moieties such as vanillic acid, syringic acid, and ferulic acid). These results support the findings of Zhou et al. [202] that did not detect ferrous ion chelating activity for the same compounds or *p*-coumaric acid using a spectrophotometric method. In contrast, ferulic and vanillic acids presented Cu^2+^-chelating capacity according to the electron spin resonance (ESR) spectrometry method [201].

Atherosclerosis is the principal CVD risk in obese patients with hepatic steatosis. According to a study by Tarantino et al. [203], altered copper bioavailability predicts early atherosclerosis. In vitro cupric ion-induced human low-density lipoprotein (LDL-c) peroxidation suggests that polyphenols may be beneficial for reducing CVD risk. The fact that ferulic acid presents a chelating capacity [202] supports metal chelation as being one of the possible mechanisms involved in the ability of ferulic acid-rich sources (e.g., wheat, rice, and other cereals) to decrease the cupric ion-induced human LDL-c peroxidation [42,204,205,206,207,208].

More recently, the antioxidant activity of ferulic acid was studied theoretically, analyzing the influence of stereochemistry and solvents [209]. The study has demonstrated that lower energy is needed for deprotonating OH groups of ferulic acid stereoisomers in polar media (dimethyl sulfoxide, ethanol, and water) compared to vacuum, which favors metal chelation when the compounds are in hydrophilic solvents. The trend of metal chelation capacity of ferulic acid dissolved in water was (increasing acidity values, decreasing chelating abilities): *trans*-ferulic acid > *cis*-ferulic acid > gallic acid > myricetin > caffeic acid > kaempferol > apigenin > kaempferol > quercetin > epicatechin > resveratrol > phenol. These analyses suggest that ferulic acid may be a metal-chelating agent. Interestingly, the ferulic acid chelation capacity reported by Sevgi et al. [190] is higher than that of gallic acid but not higher than that of caffeic acid. Therefore, partially supporting the rank suggested by Urbaniak [209] and demonstrating that more investigation is necessary.

Zhang et al. [21] verified the antioxidant potential of white, light-purple, and black rice-derived soluble and insoluble-bound phenolics using in vitro assays (DPPH and ORAC). DPPH radical scavenging activity ranged from 1.39 to 9.11 μM Trolox equivalents (TE)/g for soluble phenolics and from 0.90 to 2.25 μM TE/g for insoluble-bound phenolics, with white rice having the lowest values while the black cultivar was responsible for the highest antioxidant activity. The same trend was observed for the ORAC assay. This result is probably related to the fact that in white rice, the bran and the germ are removed during polishing, excluding the richest grain fractions in terms of phenolic compounds, thus diminishing its antioxidant capacity.

Twelve commercial wheat (Triticum aestivum) cultivars were evaluated by Podio et al. [129]. According to these authors, who also identified *cis*- and *trans*-ferulic acids in all samples, four isomeric forms of diferulic acid were among the key molecules influencing the antioxidant capacity (e.g., scavenging activity towards ABTS radical cation and ferric reducing antioxidant power). Red and white common wheat cultivars were studied by di Silvestro et al. [134]; the red grains exhibited the highest antioxidant potential, regardless of the method (FRAP or DPPH). In contrast, the reducing power was affected mainly by genotype, while the environment played the most significant role in the ability of the phenolics to scavenge DPPH.

Hu et al. [141] demonstrated a concentration-dependent inhibition by dark-blue wheat grain bran extract on hydrogen peroxide-induced intracellular oxidation. Anthocyanin-containing fruits such as grapes and other berries [210,211,212] have recently been regarded as superfruits due to their high antiradical activity [213]. Nevertheless, Hu et al. [141] stated that the cellular antioxidant activity of the pigmented wheat phenolic extract was similar to berries (Saskatoon berries and blackberries) [214,215]. The pigmented wheat extract also suppressed nitric oxide production in LPS-activated macrophages, suggesting its possible role as a source of anti-inflammatory compounds. Furthermore, solid phase extraction was employed to separate phenolic acids from anthocyanins to evaluate the contribution of each one of them to the antiradical activity. Their results demonstrated that 69% of the overall radical scavenging activity could be attributed to the anthocyanin content, while the fraction containing phenolic acids was responsible for only 19% [141].

The antiradical activity of anthocyanins can occur through hydrogen atom transfer and electron transfer. A structure–activity study [216] has shown that the presence of acyl groups in the structure of anthocyanins positively impacts the antiradical activity toward lipids due to the higher affinity. Moreover, a 3′-OCH3 group at the B ring lowers the bond dissociation energy and stabilizes the free radical, enhancing the scavenging activity.

Old and modern cultivars of common wheat (*Triticum aestivum* L.) were screened for their antiradical activity towards DPPH radical and reducing power by FRAP assay. The flavonoid content and the ratio of flavonoids/polyphenols were the main parameters influencing common wheat’s antiradical and reducing power. ROS generation is known as a proliferative stimulus in cancer cells. According to the authors [138], significant multiple correlations existed among the chemical assays (DPPH and FRAP) and intracellular protection towards ROS generation, as evaluated by principal component analysis (PCA).

Lending support to the data reported by Hu et al. [141], dose-dependent protection (5–20 µg gallic acid equivalents/mL) of phenolics from common wheat was observed in cultures of neonatal rat cardiomyocytes subjected to hydrogen peroxide-induced intracellular ROS generation [138]. The same trend was observed in leukemic cells (HL60 cell lines). These later authors also reported the cytoprotective effect of wheat phenolic extracts in neonatal rat cardiomyocytes and antiproliferative effects in wheat phenolic extract HL60 cell lines. Phenolic acids have demonstrated desirable characteristics in what concerns antioxidant capacity. Psotová et al. [217] reported that cynarin, protocatechuic, caffeic, rosmarinic, and chlorogenic acids display metal-chelating properties toward Cu (II) and Fe (II), except for protocatechuic acid. In their study, ferulic acid did not demonstrate any ability to complex with transition metals. All phenolic acids studied prevented lipid peroxidation of enterocytes in a dose-dependent manner and inhibited lipoperoxidation of rat mitochondria, with rosmarinic acid showing the highest inhibitory effect (IC50 of 0.09 mM).

The antioxidant capacity of each rice grain fraction was studied by Shao et al. [40]. The bran was reported to make the highest contribution to the antioxidant activity, followed by the germ and endosperm. Colored rice (red and black) fractions once more surpassed polished white rice in what concerns the inhibitory activity against free radicals, confirming the correlation between phenolic composition and antioxidant activity. It was reported that the bran contributed 76, 93, and 91% to the antiradical activity towards DPPH of white, red, and black rice, respectively. The ORAC assay’s corresponding contributions were 61, 91, and 88%, respectively.

Butsat et al. [39] reported that the insoluble-bound phenolics contribute more to the antioxidant capacity of rice hull than the soluble phenolics, which was verified by the DPPH, ABTS, and FRAP assays. In addition, correlational analyses showed that the most significant phenolic acids identified in rice hull (i.e., *p*-coumaric, ferulic, and gallic acids in the soluble fraction and *p*-coumaric and ferulic acids in the insoluble-bound fraction) made the highest contribution to the antioxidant potential. Such results should be confirmed by carrying out human cell lines and in vivo studies to provide information that applies to those who have experience in the digestion of phenolic compounds in wheat, rice, and other cereal grains. Some data, primarily in rice and wheat bran, are available. These aspects will be discussed in the subsequent sections.

## 7. Potential Health Effects

In a study performed in rats, with the addition of cholesterol in the diet (0.06%), rice bran (full fat), oat, barley bran, and soybean fiber were found to reduce circulating cholesterol levels. Diets containing rice bran significantly decreased the total blood cholesterol compared with the placebo; rice bran being the most effective food in reducing liver and plasma total cholesterol and increasing the high-density lipoprotein (HDL) to total cholesterol ratio [218]. A significant reduction in triglyceride levels and liver cholesterol in rats fed rice and wheat bran was observed in another study [219]. According to the authors, the positive effects in the LDL levels of mice fed a bran diet may stem from improving the function of specific receptors in the liver.

According to Chen et al. [220], polysaccharides from rice bran have been found to significantly increase the content of enzymes catalase (CAT) and superoxide dismutase (SOD) in the liver, serum, and spleen of mice while the content of malondialdehyde was lowered. The same polysaccharide [220] that is often linked to insoluble-bound phenolics, upregulated the expression of nuclear factor E2-related factor 2 (Nrf2) and its downstream antioxidant enzymes, namely, heme oxygenase (HO-1) and oxireductase 1 (NQ01). Furthermore, the activity of the antioxidant response element luciferase was enhanced. In the human body, SOD serves as a natural superoxide free radical scavenger and may contribute to reduce lipid peroxidation by converting the superoxide radical into water, while malondialdehyde in serum is an oxidative stress indicator. Therefore, the tested rice polysaccharide may have a protective effect, reducing the cardiometabolic risk.

Rats fed a high-fat and high-cholesterol diet and treated with the aqueous enzymatic extract (AEERB) from rice bran were analyzed by Wang et al. [221]. The authors observed a reduction in the atherogenic index and serum lipid levels due to AEERB administration. This effect may be related to the inhibited hepatic 3-hydroxyl-3-methylglutaryl CoA reductase activity, a metabolic pathway responsible for synthesizing cholesterol and other isoprenoids, and increased fecal excretion of total lipids and total cholesterol. As for the oxidative parameters, the dietary AEERB decreased the malondialdehyde and protein carbonyl content. Likewise, it enhanced the serum, liver, and brain antioxidant status as evidenced by increased antioxidant enzymes such as CAT, SOD, and glutathione peroxidase (GSH-Px).

According to Hou et al. [222], anthocyanin-rich black rice bran extract (ARBE) administrated as an oral solution reduced aminotransferase activities in the serum of mice treated with carbon tetrachloride. The enhancement in SOD and GSH-Px levels observed an increased antioxidant effect, while thiobarbituric acid and 8-hydroxy-2-deoxyguanosine, biomarkers of oxidative stress, had their levels reduced. The improvement in the redox homeostasis evidenced the effect of hepatoprotection against carbon tetrachloride-induced injury. Cyanidin-3-glucoside, which accounted for almost 90% of total anthocyanins in the tested samples, was suggested as the main active compound considering the results obtained using L-02 cells. According to the authors [222], black rice bran should be considered a potential functional food ingredient or source of nutraceuticals bearing hepatoprotective properties. Edrisi et al. [223] aimed to investigate the effects of rice husk powder and rice, along an energy-restricted diet, in overweight and obese adults. The rice husk and bran groups found reduced serum levels of C reactive-protein and interleukin-6.

Ferulic acid has demonstrated inhibitory activity against alpha-glucosidase, a metabolic enzyme that breaks down maltose and maltoligosaccharides into glucose units, being highly active in type 2 diabetics. A structure–activity relationship study [224] has shown that having more than one hydroxyl and methoxy group in the phenolic structure increases the enzymatic inhibition power of phenolic acids by 30–55% and by 26–45%, respectively. Meanwhile, the conjugation of phenolic acids with other compounds does not seem to affect their inhibitory activity toward alpha-glucosidase. When analyzing the kinetics of this enzyme, ferulic acid was observed to display mixed inhibition, decreasing the number of active enzymes by interfering with the shape of the active site of alpha-glucosidase. The study has also revealed that not every phenolic acid displays the same type of enzymatic inhibition. For instance, protocatechuic acid competitively inhibits alpha-glucosidase, while chlorogenic acid shows uncompetitive inhibition.

Phenolic acids can also serve as natural angiotensin-converting enzyme (ACE) inhibitors, aiding in lowering blood pressure. An in silico study revealed that the hydrophobicity of the phenoxyl ring enables hydroxycinnamic and hydroxybenzoic acids to inhibit ACE [225]. In contrast to what has been observed with alpha-glucosidase, an increased number of hydroxyl and methoxyl groups on the phenolic structure hinders the inhibitory activity due to steric hindrance. On the other hand, groups that serve as hydrogen bond acceptors, such as carboxylic and acrylic acid groups, can enhance the ACE inhibitory activity of phenolic acids. The inhibition mechanism is not completely understood yet, with evidence showing both uncompetitive and non-competitive inhibition [226].

The ferulic acid ester of wheat bran oligosaccharides, feruloyl oligosaccharides (FOs), strengthens the antioxidative capacity of rat jejunum by increased levels of CAT, SOD, and GSH-Px, as well as glutathione [227]. According to the authors, administrating FOs increases the expression levels of antioxidant-related genes (heme oxygenase-1, glutamate-cysteine ligase catalytic subunit, and glutamate-cysteine ligase modifier subunit) in the jejunum. Similar antioxidant effects were seen when Wang et al. [228] used wheat bran feruloyl oligosaccharides as a dietary supplement in lambs, increasing GSH-Px, CAT, and SOD activity and affecting performance, blood metabolites, antioxidant status, and ruminal fermentation.

Using a high-fat rat model, Junejo et al. [229] analyzed whether superfine-wheat bran would improve hyperglycemia, hyperlipidemia, and obesity. Food and energy intake was reduced in the rats treated with the superfine-wheat bran. Reductions were also seen in body weight, post-prandial glucose, triglycerides, blood and liver cholesterol, malondialdehyde, and low-density lipoprotein, while high-density lipoprotein increased. In a 6-week randomized trial with men and postmenopausal women, Vanegas et al. [186] demonstrated that substituting whole grains (composed mainly of whole wheat) positively affected gut microbiota by decreasing pro-inflammatory Enterobacteriaceae.

Ferulic acid, sinapic acid, and other hydroxycinnamic acid derivatives have been tested for inhibition against peroxidation of low-density lipoprotein (LDL). To clarify what structural features of phenolic acids determine their ability to offset LDL’s oxidative damage. Hydroxycinnamic acids bearing 4-hydroxy-3-methoxyl moieties, such as ferulic and sinapic acids, have been identified as superior in inhibiting AAPH and Cu^2+^-induced LDL peroxidation [230]. Additionally, phenolic structure modification can overcome several limitations associated with their use, such as their low bioavailability. The esterification of ferulic and caffeic acids has enhanced the antitumor activity of these compounds. Li et al. [231] synthesized alkyl esters and nitric oxide-donor derivatives of ferulic and caffeic acid, showing high cytotoxicity against human cancer cell lines.

Chen et al. [232] analyzed the potential effects of wheat bran polysaccharides (WBP) and fermented wheat bran polysaccharides (FWBP) on zebrafish growth, gut microbiota, and antioxidant status due to the presence of phenolic compounds in these foods. The results indicated that FWBP had the best growth-promoting and antioxidant effects in zebrafish, resulting in an increased expression of antioxidant-function-related genes (CAT, GST, and GST), Nrf2, and P38.

The plasma and urine of adults after a single meal of unprocessed wheat bran or refined cereal (ground white rice) were studied by Price et al. [233] in a randomized cross-over design. The experimental diet based on wheat bran increased the total plasma antioxidant potential. Furthermore, wheat bran led to higher TPC and antioxidant potential in urine than ground rice.

A recent study [98] did not find a significant anti-diabetic potential of whole wheat in vitro. In contrast, by carrying out a randomized crossover trial with type 2 diabetes adults, Aberg et al. [234] suggested that consumption of less-processed whole-grain foods, including whole wheat and brown rice, may be an important strategy to improve postprandial glycemia compared with consuming whole-grain foods where the grain particle size was further reduced through milling. It is well-accepted that whole grains contain more phenolic compounds than their refined counterparts.

Wheat bran exhibits higher antioxidant properties than the other fractions obtained upon milling due to the presence of phenolic acids and other antioxidant compounds, which can prevent DNA damage and therefore have been regarded as promising candidates in preventing or lowering the risk of cancer development [42,235]. Lending support to this assumption, an overview of animal and human intervention studies carried out with wheat bran on parameters of colorectal cancer was published by Deroover [236]. The positive results summarized by these authors in animal models include a decrease in total tumors in small and large bowel tumors, colon adenomas, colon adenocarcinomas, malignant tumors, colon polyps, and intestinal tumors. Some of them report decreased cell proliferation or no positive effect. Clearly, as in the case of potential benefits against cardiovascular ailments, type 2 diabetes, and obesity, human studies are still scarce and we are far from establishing a cause and effect.

## 8. Conclusions

Rice and wheat are two of the most important crops grown worldwide. Besides their nutritional value and economic importance, they are also sources of bioactive substances, such as phenolic compounds, which can diminish oxidative stress. Pigmented rice cultivars and the by-products of rice and wheat processing have been reported as rich sources of soluble and insoluble-bound phenolic compounds, in some cases presenting higher levels of phenolics than their edible counterparts. Both crops have been genetically improved in their phenolic composition through breeding in the last decades. Although some studies overlook the insoluble-bound fraction, many reports have demonstrated that phenolic acids in these cereals are primarily concentrated in this fraction. Diets containing foods rich in these antioxidants are related to a lower incidence of chronic ailments, such as cardiovascular diseases, diabetes, and certain types of cancer. However, their action depends on their metabolic fate and bioavailability. Studies on this topic have reported low bioavailability for some types of phenolics, especially in the insoluble-bound fraction. Nevertheless, the product of their metabolism in the colon has demonstrated beneficial effects, with more studies being necessary to understand the mechanisms behind it.

Future perspectives include addressing the contribution of derivatives of ferulic acid (e.g., dehydrodiferulic acid and dehydrotriferulic acid), several monomeric flavonoids, including apigenin, luteolin, and chrysoeriol and their derivatives. More studies focused on anthocyanins are also necessary, especially the concentrations of proanthocyanidins in wheat must be further addressed. Quantitative data obtained by state-of-the-art methods (LC and/or LC-MS^n^) must be considered by plant scientists, agronomists, food scientists, and other players involved in the wheat and rice production chain. Further work should focus on strategies to increase the bioavailability of bioactive compounds from rice, wheat, and their by-products. Moreover, the by-products should be used to produce value-added products due to their functional properties. However, human trials must be conducted to support the physiological effects that have been potentially claimed thus far.

## Figures and Tables

**Table 1:** Phenolic acids detected in rice using LC and MS-based methods.

**MF**	**Identity**	**Ion *m*/*z***	**Method**	**SI**	**Ref.**
C_7_H_6_O_3_	*p*-hydroxybenzoic acid	137.0252	UPLC-Triple/TOF-MS	(−)	[102]
C_9_H_8_O_2_	cinnamic acid	147.0438	UPLC-Triple/TOF-MS	(−)	[102]
C_8_H_8_O_3_	*p*-hydroxyphenylacetic acid	151.0402	UPLC-Triple/TOF-MS	(−)	[102]
C_7_H_6_O_4_	protocatechuic acid	153.0195	UPLC-Triple/TOF-MS	(−)	[102]
C_7_H_6_O_4_	protocatechuic acid	153.0193	HPLC-LTQ-Orbitrap-MS^n^	(−)	[103]
C_9_H_8_O_3_	*p*-coumaric acid	163.0401	HPLC-LTQ-Orbitrap-MS^n^	(−)	[103]
C_9_H_8_O_3_	*o*-coumaric acid	163.0401	HPLC-LTQ-Orbitrap-MS^n^	(−)	[103]
C_8_H_8_O_4_	vanillic acid	167.0350	HPLC-LTQ-Orbitrap-MS^n^	(−)	[103]
C_8_H_8_O_4_	vanillic acid	167.0344	UPLC-Triple/TOF-MS	(−)	[102]
C_7_H_6_O_5_	gallic acid	171.0	HPLC-LTQ-Orbitrap-MS^n^	(+)	[103]
C_7_H_6_O_5_	gallic acid	169.0870	UPLC-Triple/TOF-MS	(−)	[102]
C_9_H_8_O_4_	caffeic acid	178.9769	UPLC-Triple/TOF-MS	(−)	[102]
C_8_H_8_O_4_	protocatechuic acid ethyl ester	181.0506	HPLC-LTQ-Orbitrap-MS^n^		[103]
C_10_H_10_O_4_	ferulic acid	193.0506	HPLC-LTQ-Orbitrap-MS^n^	(−)	[103]
C_10_H_10_O_4_	ferulic acid	193.0507	UPLC-Triple/TOF-MS	(−)	[102]
C_9_H_10_O_5_	syringic acid	197.0455	HPLC-LTQ-Orbitrap-MS^n^	(−)	[103]
C_9_H_10_O_5_	syringic acid	197.8086	UPLC-Triple/TOF-MS	(−)	[102]
C_11_H_12_O_5_	sinapic acid	223.0612	HPLC-LTQ-Orbitrap-MS^n^	(−)	[103]
C_12_H_14_O_5_	3,4,5-trimethoxycinnamic acid	239.0914	HPLC-LTQ-Orbitrap-MS^n^	(+)	[103]
C_16_H_20_O_10_	Dihydroferulic acid 4-*O*-glucuronide	373.1129	HPLC-LTQ-Orbitrap-MS^n^	(+)	[103]

MF, molecular formula; SI, selected ion; (−), negative mode; (+), positive mode.

**Table 3:** Phenolic acids detected in wheat using LC and MS-based methods.

MF	Identity	Ion *m*/*z*	Method	SI	Ref.
C_7_H_6_O_2_	4-hydroxybenzaldehyde	121.0290	HPLC-ESI-TOF-MS	(−)	[131,132]
C_7_H_6_O_3_	4-hydroxybenzoic acid	137.0232	UPLC-QTOF-MS	(−)	[137]
C_9_H_8_O_2_	cinnamic acid	147.0438	UPLC-QTOF-MS	(−)	[137]
C_7_H_6_O_4_	gentisic acid	153.0179, 153.0180	UPLC-QTOF-MS	(−)	[137]
C_9_H_8_O_3_	*p*-coumaric acid	163.0400	HPLC-ESI-MS, HPLC-ESI-TOF-MS	(−)	[138,139]
C_8_H_8_O_4_	vanillic acid	167.0349, 167.0350	HPLC-ESI-TOF-MS	(−)	[132,134,138]
C_7_H_6_O_5_	gallic acid	169.0129	UPLC-QTOF-MS	(−)	[137]
C_9_H_8_O_4_	caffeic acid	179.0337	UPLC-QTOF-MS	(−)	[137]
C_10_H_10_O_4_	ferulic acid	193.0506	HPLC-ESI-TOF-MS	(−)	[132,134,138]
C_11_H_14_O_3_	zingerone	193.0857	UPLC-QTOF-MS	(−)	[137]
C_9_H_10_O_5_	syringic acid	197.0455	HPLC-ESI-TOF-MS	(−)	[132,134,138]
C_11_H_12_O_5_	sinapic acid	223.0598, 223.0612	UPLC-QTOF-MS, HPLC-ESI-TOF-MS	(−)	[131,137]
C_16_H_18_O_9_	chlorogenic acid	353.0886	UPLC-QTOF-MS	(−)	[137]
C_16_H_20_O_9_	1-*O*-feruloyl-beta-D-glucose	355.1029	UPLC-QTOF-MS	(−)	[137]
C_17_H_20_O_9_	3-feruloylquinic acid	367.1044	UPLC-QTOF-MS	(−)	[137]
C_17_H_22_O_10_	1-*O*-Sinapoyl-beta-D-glucose	385.1146	UPLC-QTOF-MS	(−)	[137]
C_20_H_18_O_8_	diferulic acid	385.0917	UPLC-QTOF-MS	(−)	[137]

MF, molecular formula; SI, selected ion; (−), negative mode; (+), positive mode.

**Table 4:** Aldehydes, coumarins, stilbenoids, monomeric flavonoids, proanthocyanidins, and lignans in wheat using HPLC and MS-based methods.

MF	Identity	Ion *m*/*z*	Method	SI	Ref.
C_8_H_8_O_3_	vanillin	151.0400	UHPLC-MS, HPLC-ESI-TOF-MS	(−)	[131]
C_15_H_10_O_4_	daidzein	255.1	HPLC-ESI-MS	(+)	[142]
C_15_H_10_O_6_	kaempferol	285.0392	UPLC-QTOF-MS	(−)	[137]
C_15_H_10_O_5_	apigenin	269.0455	HPLC-ESI-TOF-MS	(−)	[131,132]
C_18_H_16_O_4_	dasytrichone	295.1002	UPLC-QTOF-MS	(−)	[137]
C_15_H_10_O_8_	myricetin	317.0306	UPLC-QTOF-MS	(−)	[137]
C_17_H_14_O_7_	5,7,4′-trihydroxy-3′,5′-dimethoxy-flavone (tricin)	329.0666	HPLC-ESI-TOF-MS	(−)	[131,132]
C_19_H_18_O_6_	tetramethylscutellarein	341.1019	UPLC-QTOF-MS	(−)	[137]
C_20_H_18_O_6_	hinokinin	353.1030	HPLC-ESI-TOF-MS	(−)	[132,134,138]
C_20_H_22_O_6_	pinoresinol	357.1343	HPLC-ESI-TOF-MS	(−)	[132,138]
C_21_H_16_O_7_	diphyllin	379.0823	UPLC-QTOF-MS	(−)	[137]
C_21_H_22_O_8_	glycosylated pinosylvin	401.1241, 401.1242	HPLC-ESI-TOF-MS	(−)	[132,134,138]
C_22_H_26_O_8_	syringaresinol	417.4390	HPLC-ESI-TOF-MS	(−)	[132]
C_21_H_20_O_10_	isovitexin/vitexin	431.0983, 431.0985	HPLC-ESI-TOF-MS, UPLC-PDA-ESI/HRMS	(−)	[131,132,133]
C_21_H_21_O_10_^+^	pelargonidin-3-glucoside	433.2710	HPLC-ESI-TOF-MS	(−)	[131]
C_21_H_20_O_11_	orientin/isoorientin	447.3800	HPLC-ESI-TOF-MS	(−)	[132]
C_23_H_24_O_12_	glycosylated 3′,4′,5′-trihydroxy-3,7-dimethylflavone	491.1195	HPLC-ESI-TOF-MS	(−)	[132,138]
C_25_H_26_O_13_	glycosylated and acetylated 3′,4′,5′- trihydroxy-3,7-dimethylflavone	533.1300	HPLC-ESI-TOF-MS	(−)	[131,132,138]
C_26_H_30_O_12_	dalpanin	533.1714	UPLC-QTOF-MS		[137]
C_26_H_32_O_12_	pinosylvin (double glycosylation)	535.1821	HPLC-ESI-TOF-MS	(−)	[132,138]
C_26_H_28_O_14_	apigenin-6-C-arabinoside-8-C-hexoside	563.1395, 563.1406	HPLC-ESI-TOF-MS	(−)	[131,132,134,138]
C_30_H_26_O_12_	Procyanidin B	577.1351	HPLC-ESI-TOF-MS	(−)	[131,132,138]
C_27_H_30_O_14_	isovitexin-2″-*O*-rhamnoside	577.1562	HPLC-ESI-TOF-MS	(−)	[132,134]
C_26_H_28_O_15_	lucenin-1/3(luteolin-6/8-C-xyloside-8/6-C-glucoside)	579.1355	HPLC-ESI-TOF-MS	(−)	[131,138]
C_27_H_30_O_15_	vicenin-2 (apigenin-6,8-di-C-glucoside)	593.1511	HPLC-ESI-TOF-MS	(−)	[132,138]
C_28_H_32_O_15_	methylisoorientin-2″-O-rhamnoside	607.1668	HPLC-ESI-TOF-MS	(−)	[132,138]
C_28_H_34_O_15_	neohesperidin	609.1876	UPLC-QTOF-MS	(−)	[137]
C_33_H_38_O_21_	apigenin-6-C-beta-galactosyl-8-C-beta-glucosyl-O-glucuronopyranoside	769.1821	HPLC-ESI-TOF-MS	(−)	[131,132]

MF Molecular Formula; SI, selected ion; (−), negative mode; (+), positive mode.

**Table 5:** The protective effect of phenolic acids towards H_2_O_2_ + UV-induced supercoiled circular DNA damage.

	Concentration (mg/mL)				
Phenolic acid	0.002	0.004	0.008	0.016	0.032
Caffeic acid	−	+	+	+	+
Chlorogenic acid	+	+	−	−	−
Cinnamic acid	−	+	+	+	+
Ferulic acid	+	+	+	+	+
Gallic acid	−	−	−	−	−
*p*-Hydroxybenzoic acid	−	−	+	+	+
Protocatechuic acid	−	−	−	−	−
Rosmarinic acid	+	+	+	+	+
Syringic acid	−	−	−	−	−
Vanillic acid	−	+	+	+	+

Adapted from Sevgi et al. [190]. (−); no protection; (+); protective effect.

**Table 6:** Reducing power (absorbance at 700 nm), metal chelation effect (%), and total antioxidant activity by β-carotene bleaching method (%) *.

Phenolic Acid	Reducing Power **	Metal Chelation ***	β-Carotene–Linoleic Acid ***
Caffeic acid	0.619 ± 0.012	62.14 ± 0.52	96.20 ± 0.22
Chlorogenic acid	0.579 ± 0.090	41.70 ± 0.38	85.73 ± 0.34
Cinnamic acid	0.598 ± 0.020	59.44 ± 0.12	93.14 ± 0.06
Ferulic acid	0.602 ± 0.014	60.71 ± 0.82	94.06 ± 0.44
Gallic acid	0.547 ± 0.022	38.70 ± 0.72	82.30 ± 0.54
*p*-Hydroxybenzoic acid	0.611 ± 0.010	39.73 ± 1.27	95.26 ± 0.40
Protocatechuic acid	0.555 ± 0.020	26.17 ± 0.86	78.12 ± 0.07
Rosmarinic acid	0.632 ± 0.077	65.05 ± 0.71	98.92 ± 0.68
Syringic acid	0.600 ± 0.115	50.71 ± 0.65	91.94 ± 0.24
Vanillic acid	0.602 ± 0.102	61.46 ± 0.92	95.81 ± 0.17

* Adapted from Sevgi et al. [190]. Reducing power significantly and positively correlated with metal chelation (r = 0.7823, *p* < 0.01) and the β-carotene–linoleic acid assay (r = 0.9566, *p* < 0.01). Metal chelation significantly and positively correlated with the β-carotene–linoleic acid assay (r = 0.8599, *p* = 0.01). ** BHT at 0.1 mg/mL (absorbance = 2.012 ± 0.029) and BHA at 0.1 mg/mL (absorbance = 1.114 ± 0.098) were used as positive controls for reducing power. *** EDTA at 2 mg/mL was used as a positive control (chelation effect = 98.40 ± 0.35%). *** BHT at 2 mg/mL (total antioxidant capacity = 86.48 ± 1.93%) and BHA at 2 mg/mL (total antioxidant capacity = 92.14 ± 0.15) were used as positive controls for the β-carotene–linoleic acid assay. Phenolic acids were evaluated at 0.1 mg/mL (reducing power) and 2 mg/mL (metal chelation and the β-carotene–linoleic acid assay).

## Data Availability

Not applicable.

## Supplementary Materials

The following supporting information can be downloaded at: https://www.mdpi.com/article/10.3390/plants11233283/s1, Table S1: The content of soluble phenolic acids in rice using LC and MS-based methods; Table S2: The content of soluble flavonoids and proanthocyanidins in rice using LC and MS-based methods; Table S3: The content of insoluble-bound phenolics in rice using LC or LC/MS-based methods; Table S4: The content of soluble phenolic acids and flavonoids in wheat using LC and MS-based methods; Table S5: The content of insoluble-bound phenolic acids and flavonoids in wheat using LC and MS-based methods [237].

## References

[1] Brown T.A., Jones M.K., Powell W., Allaby R.G. (2009). The complex origins of domesticated crops in the Fertile Crescent. Trends Ecol. Evol..

[2] Arzani A., Ashraf M. (2017). Cultivated Ancient Wheats (*Triticum* spp.): A Potential Source of Health-Beneficial Food Products. Compr. Rev. Food Sci. Food Saf..

[3] Zaharieva M., Monneveux P. (2014). Cultivated einkorn wheat (*Triticum monococcum* L. subsp. *monococcum*): The long life of a founder crop of agriculture. Genet. Resour. Crop Evol..

[4] Black M., Bewley J., Halmer P. (2006). The Encyclopedia of Seeds: Science, Technology and Uses.

[5] Heun M., Abbo S., Lev-Yadun S., Gopher A. (2012). A critical review of the protracted domestication model for Near-Eastern founder crops: Linear regression, long-distance gene flow, archaeological, and archaeobotanical evidence. J. Exp. Bot..

[6] FAOSTAT Food and Agriculture Organization of the United Nations, Statistics Division. https://www.fao.org/statistics/en/.

[7] FAO Biannual Report on Global Food Markets. https://reliefweb.int/report/world/food-outlook-biannual-report-global-food-markets-november-2022.

[8] Huang X., Kurata N., Wei X., Wang Z.-X., Wang A., Zhao Q., Zhao Y., Liu K., Lu H., Li W. (2012). A map of rice genome variation reveals the origin of cultivated rice. Nature.

[9] Konishi S., Izawa T., Lin S.Y., Ebana K., Fukuta Y., Sasaki T., Yano M. (2006). An SNP Caused Loss of Seed Shattering during Rice Domestication. Science.

[10] Gross B.L., Zhao Z. (2014). Archaeological and genetic insights into the origins of domesticated rice. Proc. Natl. Acad. Sci. USA.

[11] Londo J.P., Chiang Y.-C., Hung K.-H., Chiang T.-Y., Schaal B.A. (2006). Phylogeography of Asian wild rice, *Oryza rufipogon*, reveals multiple independent domestications of cultivated rice, *Oryza sativa*. Proc. Natl. Acad. Sci. USA.

[12] Wang W., Mauleon R., Hu Z., Chebotarov D., Tai S., Wu Z., Li M., Zheng T., Fuentes R.R., Zhang F. (2018). Genomic variation in 3010 diverse accessions of Asian cultivated rice. Nature.

[13] Civáň P., Brown T.A. (2017). Origin of rice (*Oryza sativa* L.) domestication genes. Genet. Resour. Crop Evol..

[14] Aune D., Norat T., Romundstad P., Vatten L.J. (2013). Whole grain and refined grain consumption and the risk of type 2 diabetes: A systematic review and dose–response meta-analysis of cohort studies. Eur. J. Epidemiol..

[15] Montonen J., Boeing H., Fritsche A., Schleicher E., Joost H.G., Schulze M.B., Steffen A., Pischon T. (2013). Consumption of red meat and whole-grain bread in relation to biomarkers of obesity, inflammation, glucose metabolism and oxidative stress. Eur. J. Nutr..

[16] Zong G., Gao A., Hu F.B., Sun Q. (2016). Whole Grain Intake and Mortality From All Causes, Cardiovascular Disease, and Cancer. Circulation.

[17] Călinoiu L.F., Vodnar D.C. (2018). Whole Grains and Phenolic Acids: A Review on Bioactivity, Functionality, Health Benefits and Bioavailability. Nutrients.

[18] Shahidi F., Varatharajan V., Oh W.Y., Peng H. (2019). Phenolic compounds in agri-food by-products, their bioavailability and health effects. J. Food Bioact..

[19] Chandrasekara A., Shahidi F. (2011). Determination of antioxidant activity in free and hydrolyzed fractions of millet grains and characterization of their phenolic profiles by HPLC-DAD-ESI-MS^n^. J. Funct. Foods.

[20] De Camargo A.C., Schwember A.R., Parada R., Garcia S., Maróstica M.R., Franchin M., Regitano-d’Arce M.A.B., Shahidi F. (2018). Opinion on the Hurdles and Potential Health Benefits in Value-Added Use of Plant Food Processing By-Products as Sources of Phenolic Compounds. Int. J. Mol. Sci..

[21] Zhang H., Shao Y., Bao J., Beta T. (2015). Phenolic compounds and antioxidant properties of breeding lines between the white and black rice. Food Chem..

[22] Khoo H.E., Azlan A., Tang S.T., Lim S.M. (2017). Anthocyanidins and anthocyanins: Colored pigments as food, pharmaceutical ingredients, and the potential health benefits. Food Nutr. Res..

[23] de Camargo A.C., Schewember A.R. (2019). Phenolic-driven sensory changes in functional foods. J. Food Bioact..

[24] Ambigaipalan P., de Camargo A.C., Shahidi F. (2016). Phenolic compounds of pomegranate byproducts (outer skin, mesocarp, divider membrane) and their antioxidant activities. J. Agric. Food Chem..

[25] Ambigaipalan P., de Camargo A.C., Shahidi F. (2017). Identification of phenolic antioxidants and bioactives of pomegranate seeds following juice extraction using HPLC-DAD-ESI-MS^n^. Food Chem..

[26] de Camargo A.C., Regitano-d’Arce M.A.B., Gallo C.R., Shahidi F. (2015). Gamma-irradiation induced changes in microbiological status, phenolic profile and antioxidant activity of peanut skin. J. Funct. Foods.

[27] McCallum J.A., Walker J.R.l. (1990). Proanthocyanidins in wheat bran. Cereal Chem..

[28] Wang T., He F., Chen G. (2014). Improving bioaccessibility and bioavailability of phenolic compounds in cereal grains through processing technologies: A concise review. J. Funct. Foods.

[29] Stevenson L., Phillips F., O’Sullivan K., Walton J. (2012). Wheat bran: Its composition and benefits to health, a European perspective. Int. J. Food. Sci. Nutr..

[30] Moongngarm A., Daomukda N., Khumpika S. (2012). Chemical Compositions, Phytochemicals, and Antioxidant Capacity of Rice Bran, Rice Bran Layer, and Rice Germ. APCBEE Procedia.

[31] Shahidi F., Pinaffi-Langley A.C.C., Fuentes J., Speisky H., de Camargo A.C. (2021). Vitamin E as an essential micronutrient for human health: Common, novel, and unexplored dietary sources. Free Radic. Biol. Med..

[32] Yuldasheva N.K., Ul’chenko N.T., Glushenkova A.I. (2010). Wheat germ oil. Chem. Nat. Compd..

[33] Esa N.M., Ling T.B., Peng L.S. (2013). By-products of rice processing: An overview of health benefits and applications. J. Rice Res..

[34] Liu S., Stampfer M.J., Hu F.B., Giovannucci E., Rimm E., Manson J.E., Hennekens C.H., Willett W.C. (1999). Whole-grain consumption and risk of coronary heart disease: Results from the Nurses’ Health Study. Am. J. Clin. Nutr..

[35] Meyer K.A., Kushi L.H., Jacobs D.R., Slavin J., Sellers T.A., Folsom A.R. (2000). Carbohydrates, dietary fiber, and incident type 2 diabetes in older women. Am. J. Clin. Nutr..

[36] Parker E.D.P., Liu S.M.D.S., Van Horn L.P., Tinker L.F.P., Shikany J.M.D., Eaton C.B.M.D., Margolis K.L.M.D. (2013). The association of whole grain consumption with incident type 2 diabetes: The Women’s Health Initiative Observational Study. Ann. Epidemiol..

[37] Laddomada B., Caretto S., Mita G. (2015). Wheat Bran Phenolic Acids: Bioavailability and Stability in Whole Wheat-Based Foods. Molecules.

[38] Benisi-Kohansal S., Saneei P., Salehi-Marzijarani M., Larijani B., Esmaillzadeh A. (2016). Whole-Grain Intake and Mortality from All Causes, Cardiovascular Disease, and Cancer: A Systematic Review and Dose-Response Meta-Analysis of Prospective Cohort Studies. Adv. Nutr..

[39] Butsat S., Weerapreeyakul N., Siriamornpun S. (2009). Changes in Phenolic Acids and Antioxidant Activity in Thai Rice Husk at Five Growth Stages during Grain Development. J. Agric. Food Chem..

[40] Shao Y., Xu F., Sun X., Bao J., Beta T. (2014). Phenolic acids, anthocyanins, and antioxidant capacity in rice (*Oryza sativa* L.) grains at four stages of development after flowering. Food Chem..

[41] Sun R., Sun X.F., Wang S.Q., Zhu W., Wang X.Y. (2002). Ester and ether linkages between hydroxycinnamic acids and lignins from wheat, rice, rye, and barley straws, maize stems, and fast-growing poplar wood. Ind. Crops Prod..

[42] Liyana-Pathirana C.M., Shahidi F. (2006). Importance of insoluble-bound phenolics to antioxidant properties of wheat. J. Agric. Food Chem..

[43] Verma B., Hucl P., Chibbar R.N. (2008). Phenolic Content and Antioxidant Properties of Bran in 51 Wheat Cultivars. Cereal Chem..

[44] Shewry P.R., Piironen V., Lampi A.-M., Edelmann M., Kariluoto S., Nurmi T., Fernandez-Orozco R., Ravel C., Charmet G., Andersson A.A.M. (2010). The HEALTHGRAIN Wheat Diversity Screen: Effects of Genotype and Environment on Phytochemicals and Dietary Fiber Components. J. Agric. Food Chem..

[45] Ragaee S., Guzar I., Abdel-Aal E.S.M., Seetharaman K. (2012). Bioactive components and antioxidant capacity of Ontario hard and soft wheat varieties. Can. J. Plant Sci..

[46] Narwal S., Thakur V., Sheoran S., Dahiya S., Jaswal S., Gupta R.K. (2014). Antioxidant activity and phenolic content of the Indian wheat varieties. J. Plant Biochem. Biotechnol..

[47] Yilmaz V.A., Brandolini A., Hidalgo A. (2015). Phenolic acids and antioxidant activity of wild, feral and domesticated diploid wheats. J. Cereal Sci..

[48] Sharma M., Sandhir R., Singh A., Kumar P., Mishra A., Jachak S., Singh S.P., Singh J., Roy J. (2016). Comparative Analysis of Phenolic Compound Characterization and Their Biosynthesis Genes between Two Diverse Bread Wheat (*Triticum aestivum*) Varieties Differing for Chapatti (Unleavened Flat Bread) Quality. Front. Plant Sci..

[49] Lachman J., Martinek P., Kotíková Z., Orsák M., Šulc M. (2017). Genetics and chemistry of pigments in wheat grain—A review. J. Cereal Sci..

[50] Dwivedi S.L., Upadhyaya H.D., Chung I.-M., De Vita P., García-Lara S., Guajardo-Flores D., Gutiérrez-Uribe J.A., Serna-Saldívar S.O., Rajakumar G., Sahrawat K.L. (2016). Exploiting Phenylpropanoid Derivatives to Enhance the Nutraceutical Values of Cereals and Legumes. Front Plant Sci..

[51] Nigro D., Laddomada B., Mita G., Blanco E., Colasuonno P., Simeone R., Gadaleta A., Pasqualone A., Blanco A. (2017). Genome-wide association mapping of phenolic acids in tetraploid wheats. J. Cereal Sci..

[52] Laddomada B., Durante M., Mangini G., D’Amico L., Lenucci M.S., Simeone R., Piarulli L., Mita G., Blanco A. (2017). Genetic variation for phenolic acids concentration and composition in a tetraploid wheat (*Triticum turgidum* L.) collection. Genet. Resour. Crop Evol..

[53] Lu Y., Memon A., Fuerst P., Kizonas A., Morris C., Luthria D. (2017). Changes in the phenolic acids composition during pancake preparation: Whole and refined grain flour and processed food classification by UV and NIR spectral fingerprinting method—Proof of concept. J. Food Compos. Anal..

[54] Bravo L. (1998). Polyphenols: Chemistry, Dietary Sources, Metabolism, and Nutritional Significance. Nutr. Rev..

[55] Ma D., Li Y., Zhang J., Wang C., Qin H., Ding H., Xie Y., Guo T. (2016). Accumulation of Phenolic Compounds and Expression Profiles of Phenolic Acid Biosynthesis-Related Genes in Developing Grains of White, Purple, and Red Wheat. Front. Plant Sci..

[56] Belluco B., de Camargo A.C., da Gloria E.M., Dias C.T.D.S., Button D.C., Calori-Domingues M.A. (2017). Deoxynivalenol in wheat milling fractions: A critical evaluation regarding ongoing and new legislation limits. J. Cereal Sci..

[57] Calori-Domingues M.A., Bernardi C.M.G., Nardin M.S., de Souza G.V., dos Santos F.G.R., Stein M.d.A., Gloria E.M.d., Dias C.T.d.S., de Camargo A.C. (2016). Co-occurrence and distribution of deoxynivalenol, nivalenol and zearalenone in wheat from Brazil. Food Addit. Contam Part B.

[58] Boutigny A.-L., Barreau C., Atanasova-Penichon V., Verdal-Bonnin M.-N., Pinson-Gadais L., Richard-Forget F. (2009). Ferulic acid, an efficient inhibitor of type B trichothecene biosynthesis and Tri gene expression in *Fusarium* liquid cultures. Mycol. Res..

[59] Boutigny A.-L., Atanasova-Pénichon V., Benet M., Barreau C., Richard-Forget F. (2010). Natural phenolic acids from wheat bran inhibit *Fusarium culmorum* trichothecene biosynthesis in vitro by repressing Tri gene expression. Eur. J. Plant Pathol..

[60] Gunnaiah R., Kushalappa A.C., Duggavathi R., Fox S., Somers D.J. (2012). Integrated Metabolo-Proteomic Approach to Decipher the Mechanisms by Which Wheat QTL (*Fhb1*) Contributes to Resistance against *Fusarium graminearum*. PLoS ONE.

[61] Cahoon E.B., Hall S.E., Ripp K.G., Ganzke T.S., Hitz W.D., Coughlan S.J. (2003). Metabolic redesign of vitamin E biosynthesis in plants for tocotrienol production and increased antioxidant content. Nat. Biotechnol..

[62] Chaudhary N., Khurana P. (2013). Cloning, functional characterisation and transgenic manipulation of vitamin E biosynthesis genes of wheat. Funct. Plant Biol..

[63] Mène-Saffrané L., Pellaud S. (2017). Current strategies for vitamin E biofortification of crops. Curr. Opin. Biotechnol..

[64] Shammugasamy B., Ramakrishnan Y., Ghazali H.M., Muhammad K. (2015). Tocopherol and tocotrienol contents of different varieties of rice in Malaysia. J. Sci. Food Agric..

[65] Fritsche S., Wang X., Li J., Stich B., Kopisch-Obuch F., Endrigkeit J., Leckband G., Dreyer F., Friedt W., Meng J. (2012). A candidate gene-based association study of tocopherol content and composition in rapeseed (*Brassica napus*). Front. Plant Sci..

[66] El-Azaz J., de la Torre F., Ávila C., Cánovas F.M. (2016). Identification of a small protein domain present in all plant lineages that confers high prephenate dehydratase activity. Plant J..

[67] Diepenbrock C.H., Kandianis C.B., Lipka A.E., Magallanes-Lundback M., Vaillancourt B., Góngora-Castillo E., Wallace J.G., Cepela J., Mesberg A., Bradbury P.J. (2017). Novel Loci Underlie Natural Variation in Vitamin E Levels in Maize Grain. Plant Cell.

[68] Wang H., Xu S., Fan Y., Liu N., Zhan W., Liu H., Xiao Y., Li K., Pan Q., Li W. (2018). Beyond pathways: Genetic dissection of tocopherol content in maize kernels by combining linkage and association analyses. Plant Biotechnol. J..

[69] Strobbe S., De Lepeleire J., Van Der Straeten D. (2018). From in planta Function to Vitamin-Rich Food Crops: The ACE of Biofortification. Front. Plant Sci..

[70] Carpita N.C. (1996). Structure and biogenesis of the cell walls of grasses. Annu. Rev. Plant Physiol. Plant Mol. Biol..

[71] Scheller H.V., Ulvskov P. (2010). Hemicelluloses. Annu. Rev. Plant Biol..

[72] Grabber J.H., Hatfield R.D., Ralph J., Zoń J., Amrhein N. (1995). Ferulate cross-linking in cell walls isolated from maize cell suspensions. Phytochemistry.

[73] Fry S.C. (1986). Cross-Linking of Matrix Polymers in the Growing Cell Walls of Angiosperms. Ann. Rev. Plant Physiol..

[74] Carpita N.C., Gibeaut D.M. (1993). Structural models of primary cell walls in flowering plants: Consistency of molecular structure with the physical properties of the walls during growth. Plant J..

[75] Molinari H., Pellny T., Freeman J., Shewry P., Mitchell R. (2013). Grass cell wall feruloylation: Distribution of bound ferulate and candidate gene expression in *Brachypodium distachyon*. Front. Plant Sci..

[76] Bartley L.E., Peck M.L., Kim S.-R., Ebert B., Manisseri C., Chiniquy D.M., Sykes R., Gao L., Rautengarten C., Vega-Sánchez M.E. (2013). Overexpression of a BAHD Acyltransferase, *OsAt10*, Alters Rice Cell Wall Hydroxycinnamic Acid Content and Saccharification. Plant Physiol..

[77] Wakabayashi K., Soga K., Hoson T. (2012). Phenylalanine ammonia-lyase and cell wall peroxidase are cooperatively involved in the extensive formation of ferulate network in cell walls of developing rice shoots. J. Plant Physiol..

[78] Ma X., Xia H., Liu Y., Wei H., Zheng X., Song C., Chen L., Liu H., Luo L. (2016). Transcriptomic and Metabolomic Studies Disclose Key Metabolism Pathways Contributing to Well-maintained Photosynthesis under the Drought and the Consequent Drought-Tolerance in Rice. Front. Plant Sci..

[79] Lin C.-W., Huang L.-Y., Huang C.-L., Wang Y.-C., Lai P.-H., Wang H.-V., Chang W.-C., Chiang T.-Y., Huang H.-J. (2017). Common Stress Transcriptome Analysis Reveals Functional and Genomic Architecture Differences Between Early and Delayed Response Genes. Plant Cell Physiol..

[80] Shao Y., Bao J. (2015). Polyphenols in whole rice grain: Genetic diversity and health benefits. Food Chem..

[81] Sweeney M.T., Thomson M.J., Pfeil B.E., McCouch S. (2006). Caught Red-Handed: *Rc* Encodes a Basic Helix-Loop-Helix Protein Conditioning Red Pericarp in Rice. Plant Cell.

[82] Furukawa T., Maekawa M., Oki T., Suda I., Iida S., Shimada H., Takamure I., Kadowaki K.-i. (2007). The Rc and Rd genes are involved in proanthocyanidin synthesis in rice pericarp. Plant J..

[83] Kim C., Kikuchi S., Kim Y., Park S., Yoon U., Lee G., Choi J., Kim Y., Park S. (2010). Computational identification of seed-specific transcription factors involved in anthocyanin production in black rice. Bio. Chip J..

[84] Xu F., Bao J., Kim T.-S., Park Y.-J. (2016). Genome-wide Association Mapping of Polyphenol Contents and Antioxidant Capacity in Whole-Grain Rice. J. Agric. Food Chem..

[85] Cho M.-H., Lee S.-W. (2015). Phenolic Phytoalexins in Rice: Biological Functions and Biosynthesis. Int. J. Mol. Sci..

[86] Li W., Zhong S., Li G., Li Q., Mao B., Deng Y., Zhang H., Zeng L., Song F., He Z. (2011). Rice RING protein OsBBI1 with E3 ligase activity confers broad-spectrum resistance against Magnaporthe oryzae by modifying the cell wall defence. Cell Res..

[87] Fan S., Tian F., Li J., Hutchins W., Chen H., Yang F., Yuan X., Cui Z., Yang C.-H., He C. (2017). Identification of phenolic compounds that suppress the virulence of Xanthomonas oryzae on rice via the type III secretion system. Mol. Plant Pathol..

[88] Wang H., Hao J., Chen X., Hao Z., Wang X., Lou Y., Peng Y., Guo Z. (2007). Overexpression of rice WRKY89 enhances ultraviolet B tolerance and disease resistance in rice plants. Plant Mol. Biol..

[89] Park H.L., Lee S.-W., Jung K.-H., Hahn T.-R., Cho M.-H. (2013). Transcriptomic analysis of UV-treated rice leaves reveals UV-induced phytoalexin biosynthetic pathways and their regulatory networks in rice. Phytochemistry.

[90] Mohanty B., Lakshmanan M., Lim S.-H., Kim J.K., Ha S.-H., Lee D.-Y. (2016). Light-specific transcriptional regulation of the accumulation of carotenoids and phenolic compounds in rice leaves. Plant Signal. Behav..

[91] Amb M.K., Ahluwalia A.S. (2016). Allelopathy: Potential Role to Achieve New Milestones in Rice Cultivation. Rice Sci..

[92] Zhang Q., Li L., Li J., Wang H., Fang C., Yang X., He H. (2018). Increasing Rice Allelopathy by Induction of Barnyard Grass (*Echinochloa crus-galli*) Root Exudates. J. Plant Growth Regul..

[93] Zhang Q., Zheng X.-Y., Lin S.-X., Gu C.-Z., Li L., Li J.-Y., Fang C.-X., He H.-B. (2019). Transcriptome analysis reveals that barnyard grass exudates increase the allelopathic potential of allelopathic and non-allelopathic rice (*Oryza sativa*) accessions. Rice.

[94] Swain T., Hillis W.E. (1959). The phenolic constituents of *Prunus domestica*. I.—The quantitative analysis of phenolic constituents. J. Sci. Food Agric..

[95] Singleton V.L., Orthofer R., Lamuela-Raventós R.M. (1999). Analysis of total phenols and other oxidation substrates and antioxidants by means of folin-ciocalteu reagent. Methods Enzymol.

[96] Yu L.L., Beta T. (2015). Identification and Antioxidant Properties of Phenolic Compounds during Production of Bread from Purple Wheat Grains. Molecules.

[97] de Camargo A.C., Regitano-d’Arce M.A.B., Shahidi F. (2017). Phenolic profile of peanut by-products: Antioxidant potential and inhibition of alpha-glucosidase and lipase activities. J. Am. Oil Chem. Soc..

[98] de Camargo A.C., Silva A.P.D., Soares J.C., de Alencar S.M., Handa C.L., Cordeiro K.S., Figueira M.S., Sampaio G.R., Torres E., Shahidi F. (2021). Do Flavonoids from Durum Wheat Contribute to Its Bioactive Properties? A Prospective Study. Molecules.

[99] Yoshizawa K., Komatsu S., Takahashi I., Otsuka K. (1970). Phenolic Compounds in the Fermented Products. Agric. Biol. Chem..

[100] Jayachandran-Nair K., Sridhar R. (1975). Phenolic compounds present in rice husk. Biol. Plant.

[101] Whitmore F.W. (1974). Phenolic acids in wheat coleoptile cell walls. Plant Physiol..

[102] Ding C., Liu Q., Li P., Pei Y.S., Tao T.T., Wang Y., Yan W., Yang G.F., Shao X.L. (2019). Distribution and quantitative analysis of phenolic compounds in fractions of *Japonica* and *Indica* rice. Food Chem..

[103] Chu M.J., Liu X.M., Yan N., Wang F.Z., Du Y.M., Zhang Z.F. (2018). Partial Purification, Identification, and Quantitation of Antioxidants from Wild Rice (*Zizania latifolia*). Molecules.

[104] Hegde S., Kavitha S., Varadaraj M.C., Muralikrishna G. (2006). Degradation of cereal bran polysaccharide-phenolic acid complexes by *Aspergillus niger* CFR 1105. Food Chem..

[105] Ring S.G., Selvendran R.R. (1980). Isolation and analysis of cell wall material from beeswing wheat bran (*Triticum aestivum*). Phytochemistry.

[106] Maningat C.C., Juliano B.O. (1982). Composition of cell wall preparations of rice bran and germ. Phytochemistry.

[107] Sosulski F., Krygier K., Hogge L. (1982). Free, esterified, and insoluble-bound phenolic acids. 3. Composition of phenolic acids in cereal and potato flours. J. Agric. Food Chem..

[108] Naczk M., Shahidi F. (1989). The effect of methanol-ammonia-water treatment on the content of phenolic acids of canola. Food Chem..

[109] Klepacka J., Fornal L. (2006). Ferulic acid and its position among the phenolic compounds of wheat. Crit. Rev. Food Sci. Nutr..

[110] Zhou Z., Robards K., Helliwell S., Blanchard C. (2004). The distribution of phenolic acids in rice. Food Chem..

[111] Vichapong J., Sookserm M., Srijesdaruk V., Swatsitang P., Srijaranai S. (2010). High performance liquid chromatographic analysis of phenolic compounds and their antioxidant activities in rice varieties. LWT Food Sci. Technol..

[112] Chima J.U., Fasuan T.O. (2021). Symbiotic and adverse interplay of hypogeal germination periods on brown rice (*Oryza sativa*): Nutrient and non-nutrient characteristics. Food Prod. Process. Nutr..

[113] Xia X., Ling W., Ma J., Xia M., Hou M., Wang Q., Zhu H., Tang Z. (2006). An Anthocyanin-Rich Extract from Black Rice Enhances Atherosclerotic Plaque Stabilization in Apolipoprotein E–Deficient Mice. J. Nutr..

[114] Lu Z., Kou W., Du B., Wu Y., Zhao S., Brusco O.A., Morgan J.M., Capuzzi D.M. (2008). Effect of Xuezhikang, an Extract From Red Yeast Chinese Rice, on Coronary Events in a Chinese Population With Previous Myocardial Infarction. Am. J. Cardiol..

[115] Muntana N., Prasong S. (2010). Study on total phenolic contents and their antioxidant activities of Thai white, red and black rice bran extracts. Pak. J. Biol. Sci..

[116] Kim B., Woo S., Kim M.J., Kwon S.W., Lee J., Sung S.H., Koh H.J. (2018). Identification and quantification of flavonoids in yellow grain mutant of rice (*Oryza sativa* L.). Food Chem..

[117] Shao Y.F., Xu F.F., Sun X., Bao J.S., Beta T. (2014). Identification and quantification of phenolic acids and anthocyanins as antioxidants in bran, embryo and endosperm of white, red and black rice kernels (*Oryza sativa* L.). J. Cereal Sci..

[118] Shen Y., Jin L., Xiao P., Lu Y., Bao J. (2009). Total phenolics, flavonoids, antioxidant capacity in rice grain and their relations to grain color, size and weight. J. Cereal Sci..

[119] Laokuldilok T., Shoemaker C.F., Jongkaewwattana S., Tulyathan V. (2011). Antioxidants and Antioxidant Activity of Several Pigmented Rice Brans. J. Agric. Food Chem..

[120] Qiu Y., Liu Q., Beta T. (2010). Antioxidant properties of commercial wild rice and analysis of soluble and insoluble phenolic acids. Food Chem..

[121] Qiu Y., Liu Q., Beta T. (2009). Antioxidant Activity of Commercial Wild Rice and Identification of Flavonoid Compounds in Active Fractions. J. Agric. Food Chem..

[122] Sumczynski D., Kotásková E., Orsavová J., Valášek P. (2017). Contribution of individual phenolics to antioxidant activity and in vitro digestibility of wild rices (*Zizania aquatica* L.). Food Chem..

[123] de Camargo A.C., Lima R.S. (2019). A perspective on phenolic compounds, their potential health benefits, and international regulations: The revised Brazilian normative on food supplements. J. Food Bioact..

[124] Goufo P., Trindade H. (2014). Rice antioxidants: Phenolic acids, flavonoids, anthocyanins, proanthocyanidins, tocopherols, tocotrienols, γ-oryzanol, and phytic acid. Food Sci. Nutr..

[125] Wanyo P., Meeso N., Siriamornpun S. (2014). Effects of different treatments on the antioxidant properties and phenolic compounds of rice bran and rice husk. Food Chem..

[126] Jha P., Das A.J., Deka S.C. (2017). Optimization of ultrasound and microwave assisted extractions of polyphenols from black rice (*Oryza sativa* cv. Poireton) husk. J. Food Sci. Technol..

[127] Ramarathnam N., Osawa T., Namiki M., Kawakishi S. (1989). Chemical studies on novel rice hull antioxidants. 2. Identification of isovitexin, a C-glycosyl flavonoid. J. Agric. Food Chem..

[128] Liyana-Pathirana C.M., Shahidi F. (2007). The antioxidant potential of milling fractions from breadwheat and durum. J. Cereal Sci..

[129] Podio N.S., Baroni M.V., Wunderlin D.A. (2017). Relation between polyphenol profile and antioxidant capacity of different Argentinean wheat varieties. A Boosted Regression Trees study. Food Chem..

[130] Boukid F., Dall’Asta M., Bresciani L., Mena P., Del Rio D., Calani L., Sayar R., Seo Y.W., Yacoubi I., Mejri M. (2019). Phenolic profile and antioxidant capacity of landraces, old and modern Tunisian durum wheat. Eur. Food Res.Technol..

[131] Dinelli G., Segura Carretero A., Di Silvestro R., Marotti I., Fu S., Benedettelli S., Ghiselli L., Fernández Gutiérrez A. (2009). Determination of phenolic compounds in modern and old varieties of durum wheat using liquid chromatography coupled with time-of-flight mass spectrometry. J. Chromatogr. A.

[132] Dinelli G., Segura-Carretero A., Di Silvestro R., Marotti I., Arráez-Román D., Benedettelli S., Ghiselli L., Fernadez-Gutierrez A. (2011). Profiles of phenolic compounds in modern and old common wheat varieties determined by liquid chromatography coupled with time-of-flight mass spectrometry. J. Chromatogr. A.

[133] Geng P., Sun J., Zhang M., Li X., Harnly J.M., Chen P. (2016). Comprehensive characterization of C-glycosyl flavones in wheat (*Triticum aestivum* L.) germ using UPLC-PDA-ESI/HRMSn and mass defect filtering. J. Mass Spectrom..

[134] Di Silvestro R., Di Loreto A., Bosi S., Bregola V., Marotti I., Benedettelli S., Segura-Carretero A., Dinelli G. (2017). Environment and genotype effects on antioxidant properties of organically grown wheat varieties: A 3-year study. J. Sci. Food Agric..

[135] Yu L., Nanguet A.-L., Beta T. (2013). Comparison of antioxidant properties of refined and whole wheat flour and bread. Antioxidants.

[136] Di Loreto A., Bosi S., Montero L., Bregola V., Marotti I., Sferrazza R.E., Dinelli G., Herrero M., Cifuentes A. (2018). Determination of phenolic compounds in ancient and modern durum wheat genotypes. Electrophoresis.

[137] Santos M.C.B., Lima L.R.D.S., Nascimento F.R., Nascimento T.P.D., Cameron L.C., Ferreira M.S.L. (2018). Metabolomic approach for characterization of phenolic compounds in different wheat genotypes during grain development. Food Res. Int..

[138] Leoncini E., Prata C., Malaguti M., Marotti I., Segura-Carretero A., Catizone P., Dinelli G., Hrelia S. (2012). Phytochemical Profile and Nutraceutical Value of Old and Modern Common Wheat Cultivars. PLoS ONE.

[139] Bauer J.L., Harbaum-Piayda B., Schwarz K. (2012). Phenolic compounds from hydrolyzed and extracted fiber-rich by-products. LWT Food Sci. Technol..

[140] Gotti R., Amadesi E., Fiori J., Bosi S., Bregola V., Marotti I., Dinelli G. (2018). Differentiation of modern and ancient varieties of common wheat by quantitative capillary electrophoretic profile of phenolic acids. J. Chromatogr. A.

[141] Hu C., Cai Y.-Z., Li W., Corke H., Kitts D.D. (2007). Anthocyanin characterization and bioactivity assessment of a dark blue grained wheat (*Triticum aestivum* L. cv. Hedong Wumai) extract. Food Chem..

[142] Montevecchi G., Setti L., Olmi L., Buti M., Laviano L., Antonelli A., Sgarbi E. (2019). Determination of Free Soluble Phenolic Compounds in Grains of Ancient Wheat Varieties (*Triticum* sp. pl.) by Liquid Chromatography–Tandem Mass Spectrometry. J. Agric. Food Chem..

[143] Irakli M.N., Samanidou V.F., Biliaderis C.G., Papadoyannis I.N. (2021). Development and validation of an HPLC-method for determination of free and bound phenolic acids in cereals after solid-phase extraction. Food Chem.

[144] Hosseinian F.S., Li W., Beta T. (2008). Measurement of anthocyanins and other phytochemicals in purple wheat. Food Chem..

[145] Mattila P., Pihlava J.M., Hellstrom J. (2005). Contents of phenolic acids, alkyl- and alkenylresorcinols, and avenanthramides in commercial grain products. J. Agric. Food Chem..

[146] Zhou Z., Chen X., Zhang M., Blanchard C. (2014). Phenolics, flavonoids, proanthocyanidin and antioxidant activity of brown rice with different pericarp colors following storage. J. Stored Prod. Res..

[147] Lang G.H., Lindemann I.d.S., Ferreira C.D., Hoffmann J.F., Vanier N.L., de Oliveira M. (2019). Effects of drying temperature and long-term storage conditions on black rice phenolic compounds. Food Chem..

[148] Shahidi F., Peng H. (2018). Bioaccessibility and bioavailability of phenolic compounds. J. Food Bioact..

[149] Shahidi F., Ramakrishnan V.V., Oh W.Y. (2019). Bioavailability and metabolism of food bioactives and their health effects: A review. J. Food Bioact..

[150] Halliwell B., Zhao K., Whiteman M. (2000). The gastrointestinal tract: A major site of antioxidant action?. Free Radic. Res..

[151] Gulcin I. (2008). In vitro prooxidant effect of caffeine. J. Enzyme Inhib. Med. Chem..

[152] Carrasco-Pozo C., Speisky H., Brunser O., Pastene E., Gotteland M. (2011). Apple Peel Polyphenols Protect against Gastrointestinal Mucosa Alterations Induced by Indomethacin in Rats. J. Agric. Food Chem..

[153] Zhang J., Zheng N., Liu J., Li F.D., Li S.L., Wang J.Q. (2015). Aflatoxin B1 and aflatoxin M1 induced cytotoxicity and DNA damage in differentiated and undifferentiated Caco-2 cells. Food. Chem. Toxicol..

[154] Santili A.B.N., de Camargo A.C., Nunes R.d.S.R., Gloria E.M.d., Machado P.F., Cassoli L.D., Dias C.T.d.S., Calori-Domingues M.A. (2015). Aflatoxin M1 in raw milk from different regions of São Paulo state—Brazil. Food Addit. Contam. Part B Surveill..

[155] Vieira S., Zhang G.D., Decker E.A. (2017). Biological Implications of Lipid Oxidation Products. J. Am. Oil. Chem. Soc..

[156] de Camargo A.C., Regitano-d’Arce M.A.B., de Alencar S.M., Canniatti-Brazaca S.G., de Souza Vieira T.M.F., Shahidi F. (2016). Chemical changes and oxidative stability of peanuts as affected by the dry-blanching. J. Am. Oil. Chem. Soc..

[157] Kanner J., Lapidot T. (2001). The stomach as a bioreactor: Dietary lipid peroxidation in the gastric fluid and the effects of plant-derived antioxidants. Free Radic. Biol. Med..

[158] Zhang H., Tsao R. (2016). Dietary polyphenols, oxidative stress and antioxidant and anti-inflammatory effects. Curr. Opin. Food Sci..

[159] de Camargo C.A., Favero T.B., Morzelle C.M., Franchin M., Alvarez-Parrilla E., de la Rosa A.L., Geraldi V.M., Maróstica Júnior R.M., Shahidi F., Schwember R.A. (2019). Is chickpea a potential substitute for soybean? Phenolic bioactives and potential Health benefits. Int. J. Mol. Sci..

[160] Li L., Tsao R. (2019). UF-LC-DAD-MS^n^ for discovering enzyme inhibitors for nutraceuticals and functional foods. J. Food Bioact..

[161] Pinaffi A.C.D., Sampaio G.R., Soares M.J., Shahidi F., de Camargo A.C., Torres E. (2020). Insoluble-Bound Polyphenols Released from Guarana Powder: Inhibition of Alpha-Glucosidase and Proanthocyanidin Profile. Molecules.

[162] Zhao Z., Egashira Y., Sanada H. (2003). Digestion and absorption of ferulic acid sugar esters in rat gastrointestinal tract. J. Agric. Food Chem..

[163] Kern S.M., Bennett R.N., Mellon F.A., Kroon P.A., Garcia-Conesa M.-T. (2003). Absorption of Hydroxycinnamates in Humans after High-Bran Cereal Consumption. J. Agric. Food Chem..

[164] Konishi Y., Hagiwara K., Shimizu M. (2002). Transepithelial transport of fluorescein in Caco-2 cell monolayers and use of such transport in in vitro evaluation of phenolic acid availability. Biosci. Biotechnol. Biochem..

[165] Iftikhar M., Iftikhar A., Zhang H., Gong L., Wang J. (2020). Transport, metabolism and remedial potential of functional food extracts (FFEs) in Caco-2 cells monolayer: A review. Food Res. Int..

[166] Konishi Y., Zhao Z.H., Shimizu M. (2006). Phenolic acids are absorbed from the rat stomach with different absorption rates. J. Agric. Food Chem..

[167] Konishi Y., Shimizu M. (2003). Transepithelial transport of ferulic acid by monocarboxylic acid transporter in Caco-2 cell monolayers. Biosci. Biotechnol. Biochem..

[168] Konishi Y., Kobayashi S., Shimizu M. (2003). Transepithelial transport of *p*-coumaric acid and gallic acid in caco-2 cell monolayers. Biosc. Biotechnol. Biochem..

[169] Konishi Y., Kobayashi S. (2004). Transepithelial transport of chlorogenic acid, caffeic acid, and their colonic metabolites in intestinal Caco-2 cell monolayers. J. Agric. Food Chem..

[170] Zhao Z.H., Egashira Y., Sanada H. (2004). Ferulic acid is quickly absorbed from rat stomach as the free form and then conjugated mainly in liver. J. Nutr..

[171] Teng H., Chen L. (2019). Polyphenols and bioavailability: An update. Crit. Rev. Food. Sci. Nutr..

[172] Shahidi F., Yeo J. (2016). Insoluble-Bound Phenolics in Food. Molecules.

[173] Nordlund E., Aura A.-M., Mattila I., Kössö T., Rouau X., Poutanen K. (2012). Formation of phenolic microbial metabolites and short-chain fatty acids from rye, wheat, and oat bran and their fractions in the metabolical in vitro colon model. J. Agric. Food Chem.

[174] Rondini L., Peyrat-Maillard M.N., Marsset-Baglieri A., Fromentin G., Durand P., Tome D., Prost M., Berset C. (2004). Bound ferulic acid from bran is more bioavailable than the free compound in rat. J. Agric. Food Chem..

[175] Adam A., Crespy V., Levrat-Verny M.-A., Leenhardt F., Leuillet M., Demigné C., Ré;mésy C. (2002). The Bioavailability of Ferulic Acid Is Governed Primarily by the Food Matrix Rather than Its Metabolism in Intestine and Liver in Rats. J. Nutr..

[176] Manach C., Williamson G., Morand C., Scalbert A., Remesy C. (2005). Bioavailability and bioefficacy of polyphenols in humans. I. Review of 97 bioavailability studies. Am. J. Clin. Nutr..

[177] Janarny G., Gunathilake K.D.P.P. (2020). Changes in rice bran bioactives, their bioactivity, bioaccessibility and bioavailability with solid-state fermentation by Rhizopus oryzae. Biocatal. Agric. Biotechnol..

[178] Norkaew O., Thitisut P., Mahatheeranont S., Pawin B., Sookwong P., Yodpitak S., Lungkaphin A. (2019). Effect of wall materials on some physicochemical properties and release characteristics of encapsulated black rice anthocyanin microcapsules. Food Chem..

[179] Wijaya G.Y., Mares D.J. (2012). Apigenin di-C-glycosides (ACG) content and composition in grains of bread wheat (*Triticum aestivum*) and related species. J. Cereal Sci..

[180] Xiao J. (2017). Dietary flavonoid aglycones and their glycosides: Which show better biological significance?. Crit. Rev. Food Sci. Nutr..

[181] Angelino D., Berhow M., Ninfali P., Jeffery E.H. (2013). Caecal absorption of vitexin-2-*O*-xyloside and its aglycone apigenin, in the rat. Food Funct..

[182] Perez-Ternero C., Macià A., de Sotomayor M.A., Parrado J., Motilva M.-J., Herrera M.-D. (2017). Bioavailability of the ferulic acid-derived phenolic compounds of a rice bran enzymatic extract and their activity against superoxide production. Food Funct..

[183] de Camargo A.C., Biasoto A.C.T., Schwember A.R., Granato D., Rasera G.B., Franchin M., Rosalen P.L., Alencar S.M., Shahidi F. (2019). Should we ban total phenolics and antioxidant screening methods? The link between antioxidant potential and activation of NF-κB using phenolic compounds from grape by-products. Food Chem..

[184] Falcão H.G., Silva M.B.R., de Camargo A.C., Shahidi F., Franchin M., Rosalen P.L., Alencar S.M., Kurozawa L.E., Ida E.I. (2019). Optimizing the potential bioactivity of isoflavones from soybeans via ultrasound pretreatment: Antioxidant potential and NF-κB activation. J. Food Biochem..

[185] Shahidi F., Yeo J. (2020). Should the in vitro colorimetric assays in antioxidant and lipid oxidation evaluation be abandoned?: A critical review focusing on bioactive molecule screening assays in in vitro and in vivo models. J. Food Bioact..

[186] Yeo J., Shahidi F. (2019). Revisiting DPPH (2,2-diphenyl-1-picrylhydrazyl) assay as a useful tool in antioxidant evaluation: A new IC100 concept to address its limitations. J. Food Bioact..

[187] Shahidi F., Zhong Y. (2015). Measurement of antioxidant activity. J. Funct. Foods.

[188] Călinoiu F.L., Cătoi A.-F., Vodnar C.D. (2019). Solid-state yeast fermented wheat and oat bran as a route for delivery of antioxidants. Antioxidants.

[189] Cheng Z., Su L., Moore J., Zhou K., Luther M., Yin J.-J., Yu L. (2006). Effects of Postharvest Treatment and Heat Stress on Availability of Wheat Antioxidants. J. Agric. Food Chem..

[190] Sevgi K., Tepe B., Sarikurkcu C. (2015). Antioxidant and DNA damage protection potentials of selected phenolic acids. Food Chem. Toxicol..

[191] Park S.Y., Ha S.H., Lim S.H., Jung J.Y., Lee S.M., Yeo Y., Kim J.K. (2012). Determination of phenolic acids in Korean rice (*Oryza sativa* L.) cultivars using gas chromatography-time-of-flight mass spectrometry. Food Sci. Biotechnol..

[192] Guo W., Beta T. (2013). Phenolic acid composition and antioxidant potential of insoluble and soluble dietary fibre extracts derived from select whole-grain cereals. Food Res. Int..

[193] Chen J., Yang J., Ma L., Li J., Shahzad N., Kim C.K. (2020). Structure-antioxidant activity relationship of methoxy, phenolic hydroxyl, and carboxylic acid groups of phenolic acids. Sci. Rep..

[194] Lu Z., Nie G., Belton P.S., Tang H., Zhao B. (2006). Structure–activity relationship analysis of antioxidant ability and neuroprotective effect of gallic acid derivatives. Neurochem. Int..

[195] Graf E. (1992). Antioxidant potential of ferulic acid. Free Radic. Biol. Med..

[196] Rice-Evans C.A., Miller N.J., Paganga G. (1996). Structure-antioxidant activity relationships of flavonoids and phenolic acids (vol 20, pg 933, 1996). Free Radic. Biol. Med..

[197] Muthukumaran S., Tranchant C., Shi J., Ye X., Xue S.J. (2017). Ellagic acid in strawberry (*Fragaria* spp.): Biological, technological, stability, and human health aspects. Food Qual. Saf..

[198] Singh C.K., Siddiqui I.A., El-Abd S., Mukhtar H., Ahmad N. (2016). Combination chemoprevention with grape antioxidants. Mol. Nutr. Food Res..

[199] Ferreira I.C.F.R., Aires E., Barreira J.C.M., Estevinho L.M. (2009). Antioxidant activity of Portuguese honey samples: Different contributions of the entire honey and phenolic extract. Food Chem..

[200] Braughler J.M., Duncan L.A., Chase R.L. (1986). The involvement of iron in lipid peroxidation. Importance of ferric to ferrous ratios in initiation. J. Biol. Chem..

[201] Andjelković M., Van Camp J., De Meulenaer B., Depaemelaere G., Socaciu C., Verloo M., Verhe R. (2006). Iron-chelation properties of phenolic acids bearing catechol and galloyl groups. Food Chem..

[202] Zhou K., Yin J.-J., Yu L. (2006). ESR determination of the reactions between selected phenolic acids and free radicals or transition metals. Food Chem..

[203] Tarantino G., Porcu C., Arciello M., Andreozzi P., Balsano C. (2018). Prediction of carotid intima-media thickness in obese patients with low prevalence of comorbidities by serum copper bioavailability. J. Gastroenterol. Hepatol..

[204] Liyanapathirana C., Shahidi F. (2004). Antioxidant activity of wheat extracts as affected by in vitro digestion. Biofactors.

[205] Liyana-Pathirana C.M., Shahidi F. (2005). Antioxidant Activity of Commercial Soft and Hard Wheat (*Triticum aestivum* L.) as Affected by Gastric pH Conditions. J. Agric. Food Chem..

[206] Hu C., Zawistowski J., Ling W.H., Kitts D.D. (2003). Black rice (*Oryza sativa* L. indica) pigmented fraction suppresses both reactive oxygen species and nitric oxide in chemical and biological model systems. J. Agric. Food Chem..

[207] Chandrasekara A., Shahidi F. (2012). Antioxidant phenolics of millet control lipid peroxidation in human LDL cholesterol and food systems. J. Am. Oil Chem. Soc..

[208] Abdel-Moemin A.R. (2011). Switching to Black Rice Diets Modulates Low-Density Lipoprotein Oxidation and Lipid Measurements in Rabbits. Am. J. Med. Sci..

[209] Urbaniak A., Szelag M., Molski M. (2013). Theoretical investigation of stereochemistry and solvent influence on antioxidant activity of ferulic acid. Comput. Theor. Chem..

[210] Valdez J.C., Bolling B.W. (2019). Anthocyanins and intestinal barrier function: A review. J. Food Bioact..

[211] Robinson J.A., Bierwirth J.E., Greenspan P., Pegg R.B. (2020). Blackberry polyphenols: Review of composition, quantity, and health impacts from in vitro and in vivo studies. J. Food Bioact..

[212] King E.S., Noll A., Glenn S., Bolling B.W. (2022). Refrigerated and frozen storage impact aronia berry quality. Food Prod. Process. Nutr..

[213] Chang S.K., Alasalvar C., Shahidi F. (2019). Superfruits: Phytochemicals, antioxidant efficacies, and health effects—A comprehensive review. Crit. Rev. Food Sci. Nutr..

[214] Hu C., Kwok B.H.L., Kitts D.D. (2005). Saskatoon berries (*Amelanchier alnifolia* Nutt.) scavenge free radicals and inhibit intracellular oxidation. Food Res. Int..

[215] Elisia I., Hu C., Popovich D.G., Kitts D.D. (2007). Antioxidant assessment of an anthocyanin-enriched blackberry extract. Food Chem..

[216] Ma Y., Feng Y., Diao T., Zeng W., Zuo Y. (2020). Experimental and Theoretical Study on Antioxidant Activity of the Four Anthocyanins. J. Mol. Struct..

[217] Psotová J., Lasovský J., Vicar J. (2003). Metal-chelating properties, electrochemical behavior, scavenging and cytoprotective activities of six natural phenolics. Biomed. Pap. Med. Fac. Univ. Palacký Czech Repub..

[218] Hundemer J.K., Nabar S.P., Shriver B.J., Forman L.P. (1991). Dietary fiber sources lower blood cholesterol in C57BL/6 mice. J. Nutr..

[219] Topping D.L., Illman R.J., Roach P.D., Trimble R.P., Kambouris A., Nestel P.J. (1990). Modulation of the hypolipidemic effect of fish oils by dietary fiber in rats: Studies with rice and wheat bran. J. Nutr..

[220] Chen F., Huang S.Y., Huang G.L. (2021). Preparation, activity, and antioxidant mechanism of rice bran polysaccharide. Food Funct..

[221] Wang Y.X., Li Y., Sun A.M., Wang F.J., Yu G.P. (2014). Hypolipidemic and Antioxidative Effects of Aqueous Enzymatic Extract from Rice Bran in Rats Fed a High-Fat and -Cholesterol Diet. Nutrients.

[222] Hou F.L., Zhang R.F., Zhang M.W., Su D.X., Wei Z.C., Deng Y.Y., Zhang Y., Chi J.W., Tang X.J. (2013). Hepatoprotective and antioxidant activity of anthocyanins in black rice bran on carbon tetrachloride-induced liver injury in mice. J. Funct. Foods.

[223] Edrisi F., Salehi M., Ahmadi A., Fararoei M., Rusta F., Mahmoodianfard S. (2018). Effects of supplementation with rice husk powder and rice bran on inflammatory factors in overweight and obese adults following an energy-restricted diet: A randomized controlled trial. Eur. J. Nutr..

[224] Malunga L.N., Thandapilly S.J., Ames N. (2018). Cereal-derived phenolic acids and intestinal alpha glucosidase activity inhibition: Structural activity relationship. J. Food Biochem..

[225] Al Shukor N., Van Camp J., Gonzales G.B., Staljanssens D., Struijs K., Zotti M.J., Raes K., Smagghe G. (2013). Angiotensin-Converting Enzyme Inhibitory Effects by Plant Phenolic Compounds: A Study of Structure Activity Relationships. J. Agric. Food Chem..

[226] Alu’Datt M.H., Rababah T., Alhamad M.N., Al-Mahasneh M.A., Ereifej K., Al-Karaki G., Al-Duais M., Andrade J.E., Tranchant C.C., Kubow S. (2017). Profiles of free and bound phenolics extracted from Citrus fruits and their roles in biological systems: Content, and antioxidant, anti-diabetic and anti-hypertensive properties. Food Funct..

[227] Wang W.W., Wang Y., Duan Y.X., Meng Z.Q., An X.P., Qi J.W. (2022). Regulation of wheat bran feruloyl oligosaccharides in the intestinal antioxidative capacity of rats associated with the p38/JNK-Nrf2 signaling pathway and gut microbiota. J. Sci. Food Agric..

[228] Wang Y., Meng Z.Q., Guo J.Q., Wang W.W., Duan Y.X., Hao X.R., Wang R.F., An X.P., Qi J.W. (2019). Effect of wheat bran feruloyl oligosaccharides on the performance, blood metabolites, antioxidant status and rumen fermentation of lambs. Small Rumin. Res..

[229] Junejo S.A., Geng H.H., Li S.N., Kaka A.K., Rashid A., Zhou Y.B. (2020). Superfine wheat bran improves the hyperglycemic and hyperlipidemic properties in a high-fat rat model. Food Sci. Biotechnol..

[230] Cheng J.-C., Dai F., Zhou B., Yang L., Liu Z.-L. (2007). Antioxidant activity of hydroxycinnamic acid derivatives in human low density lipoprotein: Mechanism and structure–activity relationship. Food Chem..

[231] Li W., Li N., Tang Y., Li B., Liu L., Zhang X., Fu H., Duan J.-A. (2012). Biological activity evaluation and structure–activity relationships analysis of ferulic acid and caffeic acid derivatives for anticancer. Bioorg. Med. Chem. Lett..

[232] Chen Q.Y., Wang Y., Yin N., Wang R.F., Zheng Y., Yang Y.P., An X.P., Qi J.W. (2022). Polysaccharides from fermented wheat bran enhanced the growth performance of zebrafish (*Danio rerio*) through improving gut microflora and antioxidant status. Aquacult. Rep..

[233] Price R.K., Welch R.W., Lee-Manion A.M., Bradbury I., Strain J.J. (2008). Total phenolics and antioxidant potential in plasma and urine of humans after consumption of wheat bran. Cereal Chem..

[234] Aberg S., Mann J., Neumann S., Ross A.B., Reynolds A.N. (2020). Whole-Grain Processing and Glycemic Control in Type 2 Diabetes: A Randomized Crossover Trial. Diabetes Care.

[235] Higuchi M. (2014). Antioxidant Propertiesof Wheat Branagainst Oxidative Stress. Wheat and Rice in Disease Prevention and Health.

[236] Deroover L., Tie Y.X., Verspreet J., Courtin C.M., Verbeke K. (2020). Modifying wheat bran to improve its health benefits. Crit. Rev. Food Sci. Nutr..

[237] Bueno-Herrera M., Pérez-Magariño S. (2020). Validation of an extraction method for the quantification of soluble free and insoluble bound phenolic compounds in wheat by HPLC-DAD. J. Cereal Sci..

