# Supplementary material for: Wheat and Rice beyond Phenolic Acids: Genetics, Identification Database, Antioxidant Properties, and Potential Health Effects
Source: Plants (Basel). 2022 Nov 29;11(23):3283. doi: 10.3390/plants11233283 (PMC9739071; doi:10.3390/plants11233283)
Supplement: Supplementary file 1 [file plants-11-03283-s001.zip › plants-2036692-supplementary.pdf]

Wheat and rice beyond phenolic acids: Genetics, identification database, antioxidant properties, and potential health effects

Supplementary material

Table S1 - The content of soluble phenolic acids in rice using LC and MS-based methods

**Phenolic acid**	**Wild Rice** [1]	**Red rice** [1]	**White rice** [1]	**White**[2]	**Red**[2]	**Black**[2]
	µg/g	µg/g	µg/g	mg/100g	mg/100g	mg/100g
p-Hydroxybenzaldehyde	12.1 - 15.6	nd	nd			
p-Hydroxybenzoic acid	7.1 - 11.1	nd	0.8			
trans-Cinnamic acid	5.49	5.27				
Vanillin	13.0 - 22.3	1.0	nd			
Protocatechuic acid	12.9 - 15.6	7.8	nd	nd	nd	4.45
p-Coumaric acid	6.7 - 7.0	1.1	1.2	0.40	0.39	0.63
o-Coumaric acid	2.9 - 10.0	nd	nd			
Vanillic acid	6.3 - 17.8	nd	1.3	nd	nd	4.48
Gallic acid	64.6 - 167.1	1.1	0.2			
Protocatechuic acid ethyl ester	2.0 - 6.1	nd	nd			
Ferulic acid	121.1 - 189.7	12.4	10.9	1.11	1.60	2.07
Isoferulic acid				0.40	0.41	1.16
Syringic acid	5.1 - 19.5	nd	0.9	1.08	0.81	nd
Sinapic acid	26.8 - 59.4	3.2	4.6	nd	0.98	0.58

Abbreviations: nd, non-detected.

Table S2 - The content of soluble flavonoids and proanthocyanidins in rice using LC and MS-based methods

**Flavonoid/Proanthocyanidin**	**Wild Rice** [1]	**Red rice** [1]	**White rice** [1]		**Yellow rice**[3]	**Red rice**[4]
	μg/g	μg/g	μg/g		mg/g	μg/g
Catechin	15.6 - 21.3	6.6	nd	Isoorientin 2″-*O*- glucoside	27.46	
Epicatechin	24.3 - 43.3	3.5	nd	Luteolin 6-C-glucoside (Isoorientin)	62.46	
Quercetin	15.4 - 44.1	16.6	nd	Vitexin 2″-O-glucoside	19.65	
Epigallocatechin	7.5 - 10.0	nd	nd	Apigenin 6-C-glucoside (Isovitexin)	13.20	
Procyanidin B1	10.2 - 13.0	7.0	nd	Isoscoparin 2″-O- glucoside	22.40	
Procyanidin B2	5.0 - 5.5	2.4	nd	Chrysoeriol 6-C- glucoside (Isoscoparin)	13.66	
Procyanidin B3	6.3 - 9.4	3.4	nd			
Procyanidin C1	17.0 - 24.2	6.0	nd	Cyanidin 3-O-glucoside		55.95
Rutin	83.6 - 103.7	20.8	15.7	Peonidin 3-O-glucoside		16.46

Abbreviations: nd, non-detected.

Table S3 - The content of insoluble-bound phenolics in rice using LC or LC/MS-based methods

**Phenolic acids**	**White[2]**	**White[4]**	**Red[2]**	**Red[4]**	**Black[2]**	**Black[4]**
	**mg/100g**	**mg/100g**	**mg/100g**	**mg/100g**	**mg/100g**	**mg/100g**
Protocatechuic acid	nd	nd	0.15	1.26	0.76	4.48
p-Hydroxybenzoic acid	nd	nr	nd	nr	nd	nr
Vanillic acid	nd	nd	nd	nd	4.87	5.38
Syringic acid	0.61	0.60	0.50	0.52	0.67	0.63
p-Coumaric acid	2.68	1.36	1.83	1.05	2.06	2.00
Ferulic acid	7.79	4.28	7.74	5.73	18.57	17.92
Isoferulic acid	1.20	1.59	0.80	1.29	1.04	1.33
Sinapic acid	nd	nr	nd	nr	0.39	Nr

Abbreviations: nd, non-detected; nr, non-reported.

Table S4 - The content of soluble phenolic acids and flavonoids in wheat using LC and MS-based methods

**Phenolic acids**	**Old wheat[5]**	**Modern durum wheat[5]**	**Common wheat[6]**	**Durum wheat[7]**	**Common wheat [7]**	**Anthocyanins**	**Purple wheat [8]**
	µg/g	µg/g	mg/kg	µg/g	µg/g		mg/kg
p-Hydroxybenzaldehyde	0.068	0.033			0.43	Delphinidin 3-galactoside	38.3
p-Hydroxybenzoic acid			7.4	0.47		Delphinidin 3-arabinoside	16.7
trans-Cinnamic acid				0.60	0.91	Cyanidin 3-arabinose	25.1
Vanillin	0.284	0.088				Petunidin 3-galactoside	40.4
Protocatechuic acid			nd	nd	nd	Cyanidin 3-galactoside	72.0
p-Coumaric acid	1.704	0.666	37	0.51	0.53	Cyanidin 3-glucoside	103.0
o-Coumaric acid						Pelargonidin 3-glucoside	28.8
Vanillic acid			15	0.44	1.21	Peonidin 3-glucoside	2.7
Gallic acid				0.48	0.54	Malvidin 3-glucoside	51.6
Caffeic acid			37	0.52		Cyanidin chloride	5.2
Protocatechuic acid ethyl ester						Pelargonidin 3-galactoside	26.1
Protocatechuic acid ethyl ester						Pelargonidin 3-arabinoside	9.3
Syringaldehyde	0.632	0.399				Peonidin 3-arabinoside	28.4
Ferulic acid	126.00	61.54	890				
Isoferulic acid							
Syringic acid			13				
Sinapic acid			63				

Abbreviations: nd, non-detected.

Table S5 - The content of insoluble-bound phenolic acids and flavonoids in wheat using LC and MS-based methods

**Phenolic compounds**	**Durum wheat** [7]	**Common wheat** [7] μg/g	**Fine bran μg/g** [9]	**Coarse bran μg/g** [9]	**Flour μg/g** [9]
	μg/g				
p-Hydroxybenzoic acid	4.42	4.74	1.68	1.52	
trans-Cinnamic acid	5.49	5.27			
Vanillin			0.65	0.61	
p-Coumaric acid	11.81	13.94	9.24<br>0.69	8.05<br>0.79	0.51<br>0.15
Vanillic acid	2.95	2.97	7.25	6.52	
Gallic acid	6.04	5.95			
Ferulic acid	216.38	253.15	279<br>25.6	218<br>27.1	12.1<br>4.13
Σ DFA + TFA			56.6	39.5	2.48
Syringic acid	2.94	3.70	1.64	1.48	
Sinapic acid	41.29	45.54	7.76	9.42	0.18

Abbreviations: DFA: dehydrodiferulic acid; TFA: dehydrotriferulic acid.

References:

1. Chu, M.J.; Liu, X.M.; Yan, N.; Wang, F.Z.; Du, Y.M.; Zhang, Z.F. Partial Purification, Identification, and Quantitation of Antioxidants from Wild Rice (Zizania latifolia). Molecules 2018, 23, doi:10.3390/molecules23112782.
2. Shao, Y.F.; Xu, F.F.; Sun, X.; Bao, J.S.; Beta, T. Identification and quantification of phenolic acids and anthocyanins as antioxidants in bran, embryo and endosperm of white, red and black rice kernels (Oryza sativa L.). Journal of Cereal Science 2014, 59, 211-218, doi:10.1016/j.jcs.2014.01.004.
3. Kim, B.; Woo, S.; Kim, M.J.; Kwon, S.W.; Lee, J.; Sung, S.H.; Koh, H.J. Identification and quantification of flavonoids in yellow grain mutant of rice (Oryza sativa L.). Food Chemistry 2018, 241, 154-162, doi:10.1016/j.foodchem.2017.08.089.
4. Shao, Y.; Xu, F.; Sun, X.; Bao, J.; Beta, T. Phenolic acids, anthocyanins, and antioxidant capacity in rice (Oryza sativa L.) grains at four stages of development after flowering. Food Chemistry 2014, 143, 90-96, doi:https://doi.org/10.1016/j.foodchem.2013.07.042.
5. Di Loreto, A.; Bosi, S.; Montero, L.; Bregola, V.; Marotti, I.; Sferrazza, R.E.; Dinelli, G.; Herrero, M.; Cifuentes, A. Determination of phenolic compounds in ancient and modern durum wheat genotypes. ELECTROPHORESIS 2018, 39, 2001-2010, doi:10.1002/elps.201700439.
6. Mattila, P.; Pihlava, J.M.; Hellstrom, J. Contents of phenolic acids, alkyl- and alkenylresorcinols, and avenanthramides in commercial grain products. Journal of Agricultural and Food Chemistry 2005, 53, 8290-8295, doi:10.1021/jf051437z.
7. Irakli, M.N.; Samanidou, V.F.; Biliaderis, C.G.; Papadoyannis, I.N. Development and validation of an HPLC-method for determination of free and bound phenolic acids in cereals after solid-phase extraction. Food Chemistry 2012, 134, 1624-1632.
8. Hosseinian, F.S.; Li, W.; Beta, T. Measurement of anthocyanins and other phytochemicals in purple wheat. Food Chemistry 2008, 109, 916-924.
9. Bueno-Herrera, M.; Pérez-Magariño, S. Validation of an extraction method for the quantification of soluble free and insoluble bound phenolic compounds in wheat by HPLC-DAD. Journal of Cereal Science 2020, 93, 102984.
